# Bayesian modelling of time series data (BayModTS)—a FAIR workflow to process sparse and highly variable data

**DOI:** 10.1093/bioinformatics/btae312

**Published:** 2024-05-13

**Authors:** Sebastian Höpfl, Mohamed Albadry, Uta Dahmen, Karl-Heinz Herrmann, Eva Marie Kindler, Matthias König, Jürgen Rainer Reichenbach, Hans-Michael Tautenhahn, Weiwei Wei, Wan-Ting Zhao, Nicole Erika Radde

**Affiliations:** Institute for Stochastics and Applications, University of Stuttgart, 70569 Stuttgart, Germany; Experimental Transplantation Surgery, Department of General, Vascular and Visceral Surgery, University Hospital Jena, 07745 Jena, Germany; Department of Pathology, Faculty of Veterinary Medicine, Menoufia University, Shebin Elkom, Menoufia, Egypt; Experimental Transplantation Surgery, Department of General, Vascular and Visceral Surgery, University Hospital Jena, 07745 Jena, Germany; Medical Physics Group, Institute for Diagnostic and Interventional Radiology, University Hospital Jena, 07743 Jena, Germany; Clinic for General, Visceral and Vascular Surgery, Jena University Hospital, 07747 Jena, Germany; Institute for Biology, Faculty of Life Sciences, Humboldt-University Berlin, 10115 Berlin, Germany; Medical Physics Group, Institute for Diagnostic and Interventional Radiology, University Hospital Jena, 07743 Jena, Germany; Clinic for Visceral, Transplantation, Thoracic and Vascular Surgery, Leipzig University Hospital, 04103 Leipzig, Germany; Experimental Transplantation Surgery, Department of General, Vascular and Visceral Surgery, University Hospital Jena, 07745 Jena, Germany; Medical Physics Group, Institute for Diagnostic and Interventional Radiology, University Hospital Jena, 07743 Jena, Germany; Institute for Stochastics and Applications, University of Stuttgart, 70569 Stuttgart, Germany

## Abstract

**Motivation:**

Systems biology aims to better understand living systems through mathematical modelling of experimental and clinical data. A pervasive challenge in quantitative dynamical modelling is the integration of time series measurements, which often have high variability and low sampling resolution. Approaches are required to utilize such information while consistently handling uncertainties.

**Results:**

We present BayModTS (Bayesian modelling of time series data), a new FAIR (findable, accessible, interoperable, and reusable) workflow for processing and analysing sparse and highly variable time series data. BayModTS consistently transfers uncertainties from data to model predictions, including process knowledge via parameterized models. Further, credible differences in the dynamics of different conditions can be identified by filtering noise. To demonstrate the power and versatility of BayModTS, we applied it to three hepatic datasets gathered from three different species and with different measurement techniques: (i) blood perfusion measurements by magnetic resonance imaging in rat livers after portal vein ligation, (ii) pharmacokinetic time series of different drugs in normal and steatotic mice, and (iii) CT-based volumetric assessment of human liver remnants after clinical liver resection.

**Availability and implementation:**

The BayModTS codebase is available on GitHub at https://github.com/Systems-Theory-in-Systems-Biology/BayModTS. The repository contains a Python script for the executable BayModTS workflow and a widely applicable SBML (systems biology markup language) model for retarded transient functions. In addition, all examples from the paper are included in the repository. Data and code of the application examples are stored on DaRUS: https://doi.org/10.18419/darus-3876. The raw MRI ROI voxel data were uploaded to DaRUS: https://doi.org/10.18419/darus-3878. The steatosis metabolite data are published on FairdomHub: 10.15490/fairdomhub.1.study.1070.1.

## 1 Introduction

Biology is a field of extremes with extensive high-throughput data (e.g. omics data) on the one hand and sparse data (e.g. western blots) on the other. These data are generally characterized by high variability caused by inherent biological variation and measurement uncertainties. Due to cost and ethical aspects, small sample sizes further complicate the calculation of reliable statistics. In such a setting, it is essential to use the acquired data wisely to make progress in the field and steer biological and medical research in promising directions.

A recurring challenge in biomedical data analysis is comparing the temporal response under different conditions, i.e. comparing different time series. The current standards in biology for investigating differences in time series data are pairwise hypothesis tests, testing each time point separately (Huang *et al.* 2022, Ko *et al.* 2023) or a comparison of derived parameters such as the area under the curve, e.g. in pharmacokinetics (Scheff *et al.* 2011, Chen *et al.* 2022). Both methods are frequency-oriented and have the problem of extensive multiple testing if each time point is used as a single sample. Moreover, information about the dynamics is lost when using a summary statistic, such as the area under the curve. Methods that take the dynamics of the data into account are, e.g. semi metric ensemble time series (SMETS) (Tapinos and Mendes 2013) and the use of confidence bands (Korpela *et al.* 2014). SMETS can compare multivariate time series by measuring their pairwise distance, and nonparametric frequentistic confidence bands compute a minimum envelope of a time series but were not yet used to compare different time series.

Another challenge with sparse and noisy time series data is dealing with outliers, which can drastically distort the results. As defined by Hawkings, an outlier is an observation that deviates so strongly from other observations that it is suspected of being caused by a different mechanism (Hawkins 1980). However, the question of suspicion is not easy to answer if only a few replicates are available. The *z*-score, the Grubbs test, the Tietjen–Moore test, and Dixon’s *Q* test are existing methods for detecting single-point outliers. Further, time series filtering (e.g. bandpass filters) and dynamic linear models (Campagnoli *et al.* 2009) can smooth data series and identify outliers by considering the dynamics. We argue against outlier classification in sparse datasettings without incorporating process knowledge. The potential pitfall is a substantial information loss by excluding outliers in sparse datasettings. Using Bayesian inference with all available data, outliers are inherently corrected by incorporating process knowledge.

Fortunately, time series data are an example where knowledge of the underlying dynamics is often available. Here, we present BayModTS (Bayesian modelling of time series data), a novel findable, accessible, interoperable, and reusable (FAIR) workflow for processing time series data that incorporates process knowledge. BayModTS is designed for sparse data with low temporal resolution, a small number of replicates and high variability between replicates. BayModTS is based on a simulation model, representing the underlying data generation process. This simulation model can be an ordinary differential equation (ODE), a time-parameterized function, or any other dynamic modelling approach. For model calibration, posterior distributions of the parameters are sampled using a Bayesian approach. Ensembles of these posterior distributions are then used for forward simulations that can be compared to the data. This framework allows us to investigate whether conditions are credibly different, using measures from statistics and information theory such as credibility intervals (CIs). Importantly, BayModTS is based on systems biology modelling standards and is easily understandable and accessible for experts and novices.

We demonstrate the broad applicability of BayModTS using three datasets that investigate different characteristics of the liver in animals and humans. These data were collected using fundamentally different measurement methods: (i) quantification of blood perfusion changes in the liver lobes of rats after portal vein ligation (PVL) using magnetic resonance imaging (MRI), (ii) quantification of metabolic drug concentrations in mice via ultra-performance liquid chromatography–tandem mass spectrometry, and (iii) computed tomography (CT)-based volumetric assessment of the liver remnant in human patients after clinical liver resections (Supplement ‘Postoperative liver regeneration after resection’). We use BayModTS to statistically compare the time-series data of different conditions in these application scenarios. In particular, BayModTS deals with intra- and inter-individual variation in the studies by focusing on condition differences rather than population heterogeneity. The results are discussed in their respective biological and medical contexts.

## 2 The BayModTS workflow

**Fig. 1.**
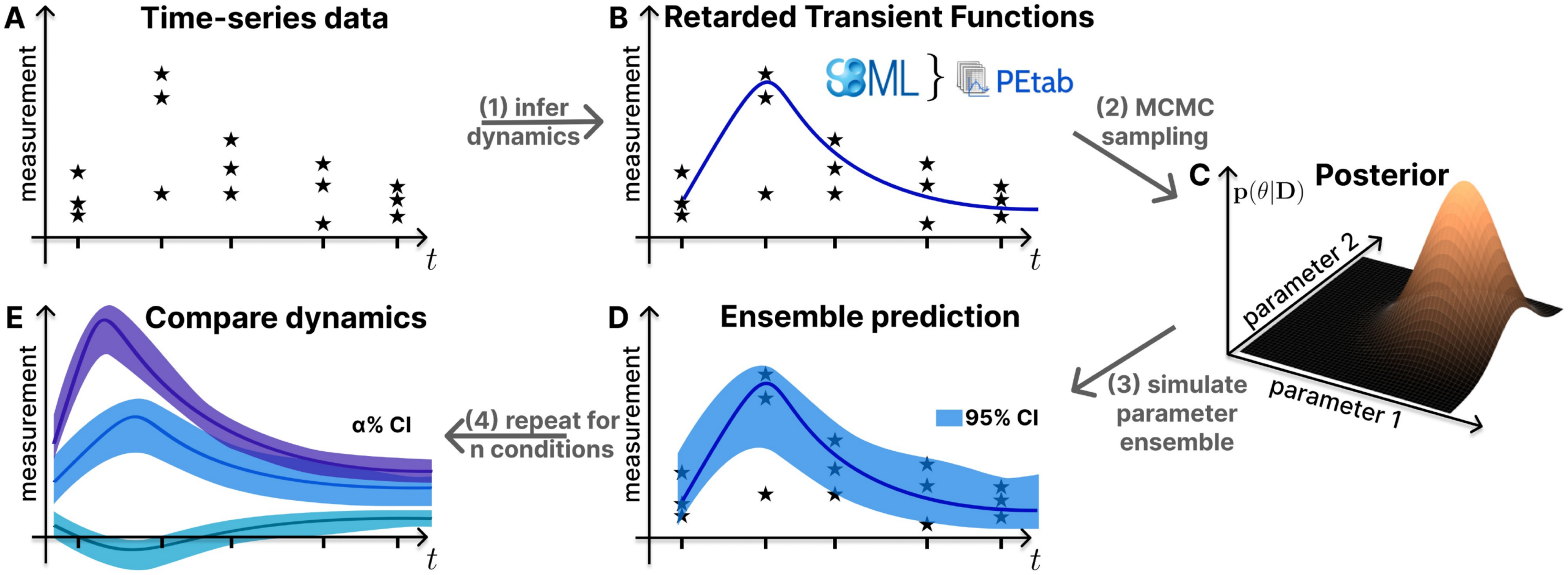
The FAIR BayModTS workflow reveals credible regions in the data space and allows statistical testing whether different datasets stem from the same data generating process. BayModTS applies a statistical Bayesian framework and a simulation model s(t,θ) to transform sparse and highly variable time series data into less noisy time courses with uncertainty estimates for model states. (A) Time series data and (B) retarded transient functions as a universal simulation model s(t,θ) are used as input together with parameter priors. (C) The Posterior distribution p(θ|D) is inferred using Markov Chain Monte Carlo sampling. (D) Simulated parameter posterior sample trajectories (Ensembles) are used to infer summary statistics that quantify model state uncertainties. We use 95% CI tubes. (E) Dynamics under different conditions can be visually compared via CI tubes.

The main applications of BayModTS are (i) to statistically test whether different datasets stem from the same data generating process and (ii) to process time series data and create continuous input functions with uncertainties for other models. In brief, BayModTS infers the dynamics of time series data via Retarded Transient Functions (RTFs) (Fig. 1, step 1). Bayesian parameter estimation via Markov Chain Monte Carlo (MCMC) sampling is used to infer the posterior distribution, given the model and the data (Fig. 1, step 2). Parameter ensembles are simulated from the posterior distribution to transfer the uncertainty from the parameter to the data space (Fig. 1, step 3). These steps are repeated for *n* conditions to compare their dynamics (Fig. 1, step 4). A more detailed description of the workflow follows.

Inputs to BayModTS are (i) serial data $D=\{m^{\left(j\right)}(t_{k})\}$, with time points k=1,…,T which do not have to be equidistant, and j=1,…,N replicates per time point (Fig. 1A), (ii) an associated simulation model s(t,θ) with parameters θ (Fig. 1B), and (iii) a prior distribution p(θ) on the model parameters.

The simulation model *s* is defined in SBML (systems biology markup language) (Keating *et al.* 2020) and leverages PEtab (Schmiester *et al.* 2021) to integrate the experimental data and an error model. BayModTS uses the explicit RTF (Kreutz 2020)

(1) $$f_{TF}(t)=\underset{\text{sustained response}}{\underset{︸}{A_{\text{sus}}\left(1-e^{-\frac{t}{t_{1}}}\right)}}\;+\;\underset{\text{transient response}}{\underset{︸}{A_{\text{trans}}\left(1-e^{-\frac{t}{t_{11}}}\right)e^{-\frac{t}{t_{2}}}}}\;+\;p_{0}$$

with transformed time

(2) $$t={\;\text{log}\;}_{10}\left(10^{t_{\text{real}}\cdot \frac{10}{T_{\text{range}}}}\;+\;10^{T_{\text{shift}}}\right)-{\;\text{log}\;}_{10}\left(1\;+\;10^{T_{\text{shift}}}\right)$$

as a universal simulation model s(t,θ) with parameters

(3) $$\theta =(A_{\text{sus}},t_{1},A_{\text{trans}},t_{11},t_{2},T_{\text{shift}},p_{0}).$$

Equation (1) consists of a transient and a persistent activation term. RTFs can describe the responses of many biological processes to various inputs without knowing the system’s detailed interactions a priori. RTF responses can be delayed by a shift parameter $T_{\text{shift}}$ via a nonlinear time transformation that leaves t=0 invariant (Equation 2). An immediate response corresponds to $T_{\text{shift}}=-2$ (Kreutz 2020), which is a valid assumption for all examples in this paper and was therefore fixed a priori for all application examples. The parameter $T_{\text{range}}$ is fixed to the range of the observation interval. Even if the system under investigation can be described by an ODE model, RTFs can serve as computationally advantageous surrogate models because they can be evaluated directly without numerical integration.

PEtab is a reproducible format for parameter estimation problems. It contains information about the estimated parameters θ, the experimental conditions, the observed data *D* and the error model needed to define the likelihood function $L_{D}(\theta )$. Here, we assume additive, independent, and identically distributed (i.i.d.) Gaussian measurement errors with equal variances $\sigma^{2}$ for all measurements,

(4) $$m(t_{k})=s(t_{k},\theta )\;+\;\epsilon \;\epsilon ∼N(0,\sigma^{2}),$$

leading to

(5) $$\begin{array}{c} L_{D}(\theta )=\prod_{k=1}^{T}\prod_{j=1}^{N}\frac{1}{\sqrt{2\pi }\sigma }\;\text{exp}\;\left(-\frac{{\left(s(t_{k},\theta )-m^{\left(j\right)}(t_{k})\right)}^{2}}{2\sigma^{2}}\right). \end{array}$$

The variance $\sigma^{2}$ can be treated as an additional unknown parameter that has to be estimated in the inverse problem or set to a fixed value for each data point or measurement series. Different approaches exist for formulating an appropriate error model and choosing hyperparameters for this model (Kreutz *et al.* 2007, Thomaseth and Radde 2023). Our pragmatic approach pools all data for estimating σ, assuming that the noise is mainly determined by the measurement method and does not vary much between time points. PEtab can be used for maximum likelihood estimation or sampling-based approaches. Here, we infer parameters from the posterior distribution (Fig. 1C)

(6) p(θ|D)∝p(D|θ)·p(θ)

in a statistical Bayesian setting to quantify the uncertainty in the parameter space. Inference is based on MCMC sampling techniques to generate posterior samples $\theta^{\left(i\right)}$, i=1,…,P (Fig. 1, step 2). For RTFs, uniform priors with wide bounds around the measurement data can be used (Kreutz 2020) and adapted for other scenarios.

Posterior predictive distributions

(7) $$p(\tilde{D}|D)=\int p(\tilde{D},\theta |D)d\theta$$

propagate the uncertainty from the parameter into the data space. Using factorization of the joint density $p(\tilde{D},\theta |D)$ and exploiting that the posterior predictive distribution (PPD) of any dataset $\tilde{D}$ is independent of *D* given the model parameters, we can reformulate Equation (7),

(8a) $$p(\tilde{D}|D)=\int p(\tilde{D}|\theta ,D)p(\theta |D)d\theta$$

(8b) $$\begin{array}{c} =\int p(\tilde{D}|\theta )p(\theta |D)d\theta . \end{array}$$

Using the posterior samples $\theta^{\left(i\right)}$, these PPDs can be estimated via Monte Carlo integration,

(9) $$p(\tilde{D}|D)\approx \frac{1}{P}\sum_{i=1}^{P}p(\tilde{D}|\theta^{\left(i\right)}).$$

A lower bound for the variability of the PPD at time *t* can be obtained by a posterior parameter ensemble prediction of simulation model trajectories s(t,θ) evaluated with posterior samples $\theta^{\left(i\right)}$,

(10) $$s^{\left(i\right)}(t)=s(t,\theta^{\left(i\right)}),$$

without adding measurement noise. Summary statistics derived from these sample trajectories represent the remaining uncertainties of the model states s(t,θ). In BayModTS, (1-α)·100% CI tubes of the model states are used to quantify the uncertainty. CIs are obtained for each time point by calculation of the percentile ranges $\left[z_{\frac{\alpha }{2}},z_{1-\frac{\alpha }{2}}\right]$ from all sample trajectories $s^{\left(i\right)}(t)$ (Fig. 1, step 3). The point-wise CIs are linearly interpolated for visualization and can be interpreted as model-informed noise filters. Data points far outside those tubes might be seen as suspicious points that need further investigation or can be classified as outliers based on the model assumption. The procedure can be repeated if the dataset includes time courses from multiple conditions. CI tubes can be used to compare dynamics under different conditions (Fig. 1, step 4 and 1E).

Regarding FAIR principles, BayModTS is interoperable and reusable using the PEtab format, which contains all information about the inference problem. Findability and accessibility are ensured by uploading the analysis results to an open public repository and assigning a digital object identifier (DOI). We provide a PEtab file containing the RTF SBML model and executable code for the Bayesian analysis on GitHub (https://github.com/Systems-Theory-in-Systems-Biology/BayModTS). Both can be easily adapted for either more complex models, different conditions, and individual data. The posterior is sampled via algorithms of the PyPesto toolbox (Schälte *et al.* 2023). PyPesto is well received and maintained by the systems biology model community and includes, besides others, an adaptive Metropolis-Hastings algorithm, a parallel tempering algorithm, and a wrapper to the ensemble sampler emcee. We recommend examining the sampling convergence via measures such as the effective sample size (ESS), visually via traces, or via the Gelman–Rubin diagnostic for multiple chains.

## 3 Application examples

*Biological context:* Hepatocellular carcinoma (HCC) is among the leading causes of cancer-related deaths worldwide and has the fastest-growing number of cancer-related deaths in the United States (El-Serag and Rudolph 2007). Still, Africa and Asia are showing the highest incidences of HCC as Hepatitis B and C are most prevalent in these continents and the leading cause of chronic liver diseases and HCC (Ferlay *et al.* 2015). Based on the evidence, liver resection is the most promising treatment option for HCC, but randomized phase III trials are ongoing (Vogel *et al.* 2018, Elderkin *et al.* 2023). However, liver resections are complex and pose the risk of major complications (Abreu *et al.* 2020). Portal vein embolization (PVE), the clinical pendant to PVL in animal models, is often performed to induce growth of the future liver remnant. In PVE, the portal influx of diseased liver lobes is ligated, leading to hypoperfusion and undersupply of nutrients in these lobes. In contrast, the nonligated lobes are hyperperfused, inducing regeneration and hypertrophy. However, the relationship between the liver mass gain of the nonligated liver lobes and liver function is still poorly understood (Christ *et al.* 2021). Moreover, comorbidities such as a high degree of hepatic steatosis might impair the proper functioning and recovery of the remaining liver volume after resection (Veteläinen *et al.* 2007).

### 3.1 Quantification of blood perfusion changes in the liver lobes of rats after PVL

**Fig. 2.**
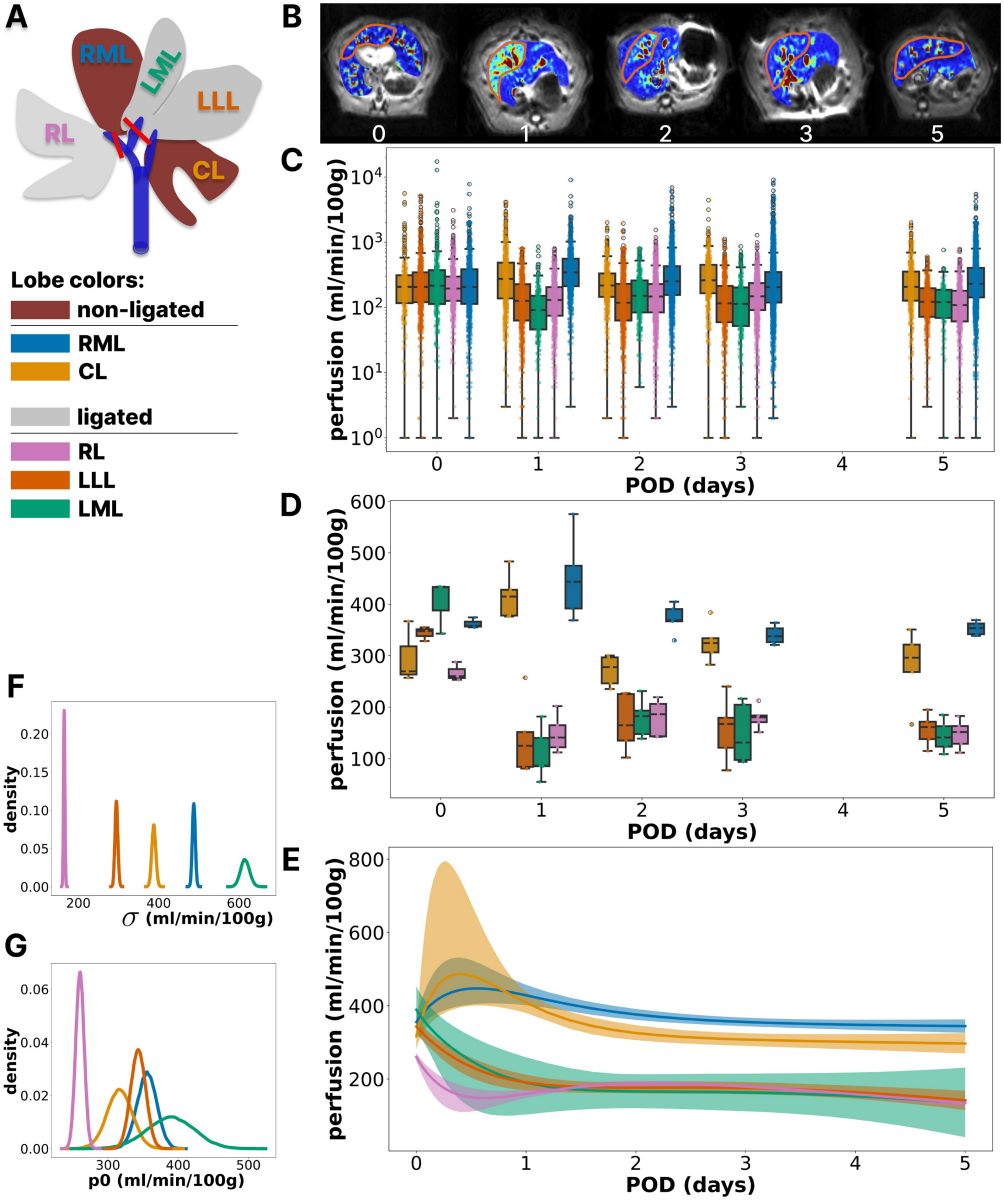
BayModTS analysis calculates perfusion courses in ligated and nonligated liver lobes from MRI data after PVL. (A) Illustration of ligated (hypoperfused) and nonligated (hyperperfused) lobes of a rat liver subjected to PVL. (B) Exemplary MRI images for 0, 1, 2, 3, and 5 POD. The outlined area (orange) refers to the ROI annotation for RML. Perfusion is colour-coded, with yellow to red values indicating high values. (C) Boxplots of individual voxel values in the selected ROIs of n=5 animals per time point (raw data). (D) State-of-the-art visualization of MRI ROI data. Boxplots are based on per-animal averaged ROI voxel data. (E) BayModTS ensemble perfusion predictions with median (solid lines) and 95% CI tubes for all lobes. (F, G) Marginal distributions of the parameters σ and $p_{0}$ per lobe, restricted to the 95% highest density interval.

This study explored *in vivo* liver perfusion in rats’ ligated and nonligated liver lobes. In total, 25 rats were subjected to 60% PVL. The experimental procedure involved ligation of the left stem of the portal vein supplying the left median lobe (LML) and left lateral lobe (LLL) and the right stem supplying the right lobe (RL) (Fig. 2A). As a result, the remaining nonligated caudate lobe (CL) and right median lobe (RML) of the liver were hyperperfused. BayModTS was used to compare the perfusion dynamics of the different lobes and to calculate continuous perfusion changes over time with uncertainties. The RTF with an immediate response was used to describe perfusion changes over time.

Changes in local tissue perfusion over time were monitored and quantified using MRI in a cross-sectional study design. Perfusion was quantitatively assessed at the voxel level. A voxel is an MRI image pixel representing a defined volume’s perfusion. The voxel has the same *xy*-axis size as the pixel, while the *z*-axis corresponds to the slice thickness. Voxels were assessed using arterial spin labelling (ASL) (Jahng *et al.* 2014), which is based on labelling spins of arterial blood by pulsed inversion and subtraction of the resulting image from a control image acquired without labelling. Negative values resulting from this subtraction were excluded as they have no physiological relevance.

Regions of interest (ROIs) that are representative of the perfusion were defined for each liver lobe. ROI selection is shown exemplary for the RML (Fig. 2B). The increased perfusion observed on postoperative day one (POD1) in the RML (Fig. 2B second picture) is visually recognizable by the increasing yellow to red colouration in the annotated region. Voxel-level perfusion values within the ROI of each lobe are shown in Fig. 2C for the different observation times. The observed variability in individual voxel values is large due to blood flow and pulsation in larger vessels. Further reasons for the high variability are that different ROIs differ in the number and size of vessels they contain and the biological variation between animals. This variability makes it difficult to identify a consistent trend over time by visually inspecting these raw data.

The current standard processing of MRI ROI data is averaging the individual voxel data per liver lobe in the animals and visualizing it as box plots (Fig. 2D). Inspection of the averaged data shows that the nonligated lobes (i.e. RML and CL) were transiently hyperperfused on POD1 and POD2, whereas perfusion in the ligated lobes decreased after POD1.

BayModTS processing of the individual voxel data results in time-continuous and smooth CI tubes (Fig. 2E), considerably reducing the variability, and revealing that the highest uncertainty is between POD0 and POD1. The CI tubes provide a credible and clear visual distinction between the dynamics of the hyper- and the hypoperfused lobes. For all three ligated lobes (LML, LLL, RL), the average perfusion values decrease by about 30%–50% within one day and remain at low values. At the same time, the nonligated lobes (RML, CL) are hyperperfused, which is most pronounced at POD1. The median perfusion of CL is already back to pre-PVL levels by POD2, which occurs more slowly in the RML. Overall, the BayModTS analysis shows that the liver adapted to ligation-induced perfusion changes within two days.

The LML showed the greatest variability in perfusion compared to the other liver lobes. Reasons for the increased uncertainty of the LML are its proximity to the heart, causing motion that affects measurements, and the putative development of collaterals leading to alternative portal inflow. In addition, the heart, liver and lung tissues are not equally susceptible to the magnetic field, distorting the MRI images. As a result, slice positions close to the lungs and heart, such as the LML, usually have poor image quality. Consequently, cardiac motion and B0 inhomogeneities reduce the usable ROI size, increasing the uncertainty reflected in the CI tubes’ size. Consistent with this, the 95% CI of the noise parameter σ (Fig. 2F) is also the largest for LML, reflecting higher measurement noise.

Conditions can also be compared by looking at the marginal posterior distributions of the model parameters. This is exemplified by the marginal posterior distributions of the parameter $p_{0}$, which describes the preoperative perfusion in each of the lobes (Fig. 2G). Here again, the LML $p_{0}$ marginal distribution is the broadest, consistent with an overall increased uncertainty of the CI tubes. The preoperative perfusion of the RL is credibly lower than that of all other lobes (the upper limit of the RL CI is below the lower limit of all other CIs). Here, the relatively low RL perfusion is likely an artefact of having fewer vessels in the typical RL slice, which disappears in the postoperative measurements.

In conclusion, BayModTS provided a continuous and credible prediction of postoperative liver perfusion in the different lobes after PVL. The BayModTS inferred CI tubes are credibly different between ligated and nonligated lobes for all postoperative days. The credibility bounds allowed quantitative hypotheses about the timing of hyperperfusion and the time scales of adaptation. Furthermore, the continuity of the BayModTS results allows the PVL data to be used as input for physiological models. In the future, this information could be valuable for determining the correct timing of surgery in human patients without the need for repeated MRI scans.

### 3.2 Influence of steatosis on drug metabolization dynamics in mice

Hepatic steatosis is a common liver disease affecting up to 25% of the population in the Western world (Younossi *et al.* 2023). However, the effects of hepatic steatosis on drug metabolism remain poorly understood. Here, we apply BayModTS to investigate statistically if different steatosis degrees in mice change the metabolization dynamics of test drugs.

**Fig. 3.**
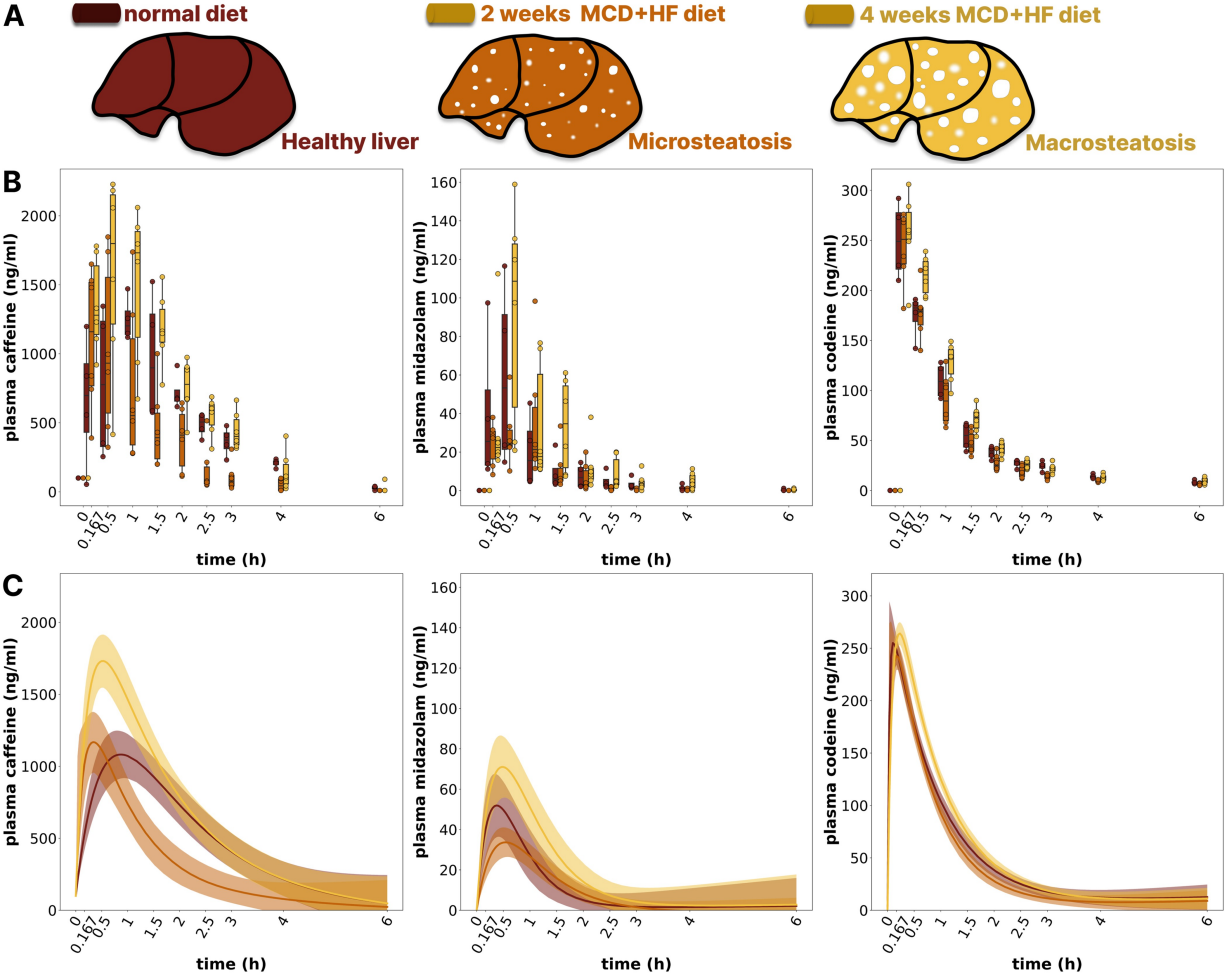
Effect of severity and pattern of periportal steatosis on pharmacokinetics. (A) Mice were fed a normal diet for 2 weeks and a MCD and HF diet for 2 or 4 weeks. The MCD+HF diet induces hepatic microsteatosis after 2 weeks and macrosteatosis after 4 weeks. (B) Boxplots of plasma drug elimination time courses. The control group (red) consisted of *n* = 4 animals, while the 2 weeks (orange) and 4 weeks (yellow) groups consisted of *n* = 6 animals each. (C) Median ensemble prediction (dark lines) and 95% CI tubes of the BayModTS analysis with RTFs.

The study by Albadry *et al.* (2022) aimed to investigate the influence of different degrees of hepatic steatosis on the pharmacokinetics of several test drugs. Mice were fed a high-fat (HF) methionine-choline deficient (MCD) diet for 2 or 4 weeks, resulting in micro- and macrosteatosis (Fig. 3A). A drug cocktail consisting of codeine, caffeine and midazolam was administered, and whole blood samples were analysed to determine drug concentrations. Peak concentrations for all three test drugs occurred within 15–60 min, followed by elimination of the drug within 4–6 h. The time course measurements showed high inter-individual variability (Fig. 3B), with time-varying variances within a condition, which made it challenging to compare conditions over the entire period.

We used BayModTS to assess the elimination dynamics of all substrates as a function of steatosis degree. In the original study (Albadry *et al.* 2022), this was accomplished by assuming an exponential decay kinetics that mimics drug degradation, while we use the RTF with immediate response (Equation 1) here. The advantages of the RTFs compared to the exponential decay kinetics are thereby (i) a more flexible model approach that also allows dynamics to a different steady-state than the initial condition and (ii) a direct evaluation without numerical integration, remarkably improving the computation time. The uncertainty in the BayModTS inferred model states is considerably smaller than the variability of the data (Fig. 3C). Our analysis suggests that macrovesicular periportal steatosis (4 weeks) delayed the clearance of all test drugs. Furthermore, peak concentrations of caffeine and midazolam were higher in macrovesicular steatosis. To validate the RTF dynamics in this example, we created a mechanistic two-compartment pharmacokinetic model for drug metabolization and repeated the BayModTS analysis with this model. The predicted medians and CI tubes for the RTF and PK approaches were similar, validating the RTF dynamics for this example (Supplementary Fig. S2).

Albadry *et al.* (2022) tested the differences of the steatosis conditions by calculating the area under the curve for each time series replicate. The four control, six 2 weeks MCD+HF, and six 4 weeks MCD+HF diet area under the curve values were analysed by a one-way ANOVA. The ANOVA calculated significant differences between the 2 and 4 weeks conditions for caffeine, midazolam and codeine. However, no significant difference between the control and the 4 weeks condition was detected (Albadry *et al.* 2022). ANOVA calculation on four to six data points only is critical. BayModTS improves the statistical analysis substantially as (i) using the area under the curve as summary statistics can disguise differences in the metabolization dynamics if different effects compensate for each other over time, (ii) BayModTS takes all data points of the replicates into account, and (iii) BayModTS provides a visual comparison of the dynamics over time.

In summary, BayModTS helped to understand the data regarding differences in pharmacokinetics and indicated an impaired metabolization in mice with macrosteatosis.

## 4 Discussion

We have introduced BayModTS, a novel workflow for processing time series data using a FAIR Bayesian analysis. BayModTS can be applied in scenarios with sparse and noisy data. The processing is based on a simulation model that can mimic the measured time series. The statistical Bayesian framework enables a consistent transfer of data variability to uncertainties in model parameters, thereby acting as a noise filter and reducing the impact of potential outliers. BayModTS can process data in areas where purely data-driven approaches fail. The BayModTS-derived dynamics can be used to compare conditions or as time-continuous input equipped with uncertainty for computational models. Our examples show broad applicability in different contexts and for different kinds of data. Importantly, BayModTS contributes to reproducibility according to the FAIR principles by using established tools of the systems biology community and adhering to standard reporting guidelines (Tiwari *et al.* 2021).

The RTFs are a flexible and computationally efficient simulation model which does not require knowledge of the underlying process. However, RTFs have two major limitations: (i) only one activation peak, and (ii) no oscillations can be modelled (Kreutz 2020). Further process knowledge is not incorporated in the model, which can lead to larger uncertainties than in mechanistic models. We showed that RTFs describe the underlying dynamics of drug metabolization in steatotic mice as well as a mechanistic pharmacokinetic model. We further demonstrate the flexibility of BayModTS with respect to user-defined SBML models by exchanging the RTF with a pharmacokinetic model incorporating domain-specific knowledge.

Properly describing observed data distributions in connection with deterministic simulation models is tricky in Bayesian settings. Forward simulations with posterior samples can filter noise because the model assumption restricts the uncertainty. Noise filtering works particularly well if the simulation model is a good description of the underlying process or is flexible enough to adapt to the course of the data. Part of the noise filtering property of BayModTS is based on the standard Likelihood (Equation 5) we use, which penalizes distances quadratically. Therefore, the uncertainty of the CI tubes of the application examples was smaller than the uncertainty in the data. In datasets having extreme values to higher and lower values simultaneously, penalizing the quadratic distance leads to regression towards the centre. The regression towards the centre is because the smaller distance to the one outlier cannot compensate for a disproportionately large distance to the other outlier. In cases where the measurement methods generate a lot of noise or a good estimator for the average population behaviour is to be found, this filter property can be beneficial. In BayModTS, the derived CI tubes are influenced by the choice of the error model, its hyperparameters and the prior. The CI tubes are currently calculated based on percentile intervals of the posterior parameter ensemble predictions. The percentile intervals cut the lower and upper α/2% of the ensemble predictions per evaluation time point, and upper and lower interval bounds are connected to form the credibility tube. Depending on the process and shape of the posterior distribution, percentile ensemble-based CIs can lead to broader or smaller credibility tubes compared to CI calculation via the highest posterior density. In the long run, calculating the credibility tubes on the posterior level would be desirable.

If only limited information about the parameters is known a-priori, noninformative priors can be used. In practice, sampling procedures and optimization algorithms usually require boundaries, which must be adjusted manually. These bounds should not restrict the sampling, which can be difficult with correlated or sloppy parameters. Model reduction techniques can be used here to improve the identifiability of the simulation model parameters (Eisenkolb *et al.* 2020).

Extensions of the workflow are easily possible for future applications. First, the method can also be applied to nontime series data. If there is only one dependent variable and serial data with known underlying dynamics, BayModTS can quantify the uncertainty and predict a credible range of the dynamics. To use serial nontime data, the user must define another dependent variable in the SBML file and include the corresponding data in the PEtab measurement table. Second, BayModTS can be applied to user-defined SBML models as demonstrated with the pharmacokinetic model for the steatosis data. Third, the BayModTS analysis allows a time-continuous description of the experimental data with uncertainty. In the future, these time-continuous functions can be used as input modules for other computational models. For example, BayModTS-derived perfusion changes after PVL could be used as input for other liver models, thereby relating liver perfusion to liver function. Fourth, conditions can be compared on the parameter level. Some parameters might differ between conditions on the parameter level, others may not. This is different when looking at the posterior parameter ensemble prediction, which shows differences in the joint parameter level. Fifth, BayModTS can also compare different processes rather than different conditions. Here, different SBML models describing the underlying processes are used for comparison.

## Supplementary Material

btae312_Supplementary_Data
