# Supplementary material for: Bayesian modelling of time series data (BayModTS)—a FAIR workflow to process sparse and highly variable data
Source: Bioinformatics. 2024 May 13;40(5):btae312. doi: 10.1093/bioinformatics/btae312 (PMC11128094; doi:10.1093/bioinformatics/btae312)
Supplement: btae312_Supplementary_Data [file btae312_supplementary_data.pdf]

Bayesian Modeling of Time Series Data (BayModTS) - A FAIR Workflow to Process Sparse and Highly Variable Data

– Supplementary Information –

These supplements contain (i) an additional BayModTS application for clinical liver regeneration data, (ii) validation of the steatosis example with a pharmacokinetic model instead of Retarded Transient Functions (RTF), (iii) the MCMC parameter sampling scatters.

Postoperative liver regeneration after resection - 3rd application example

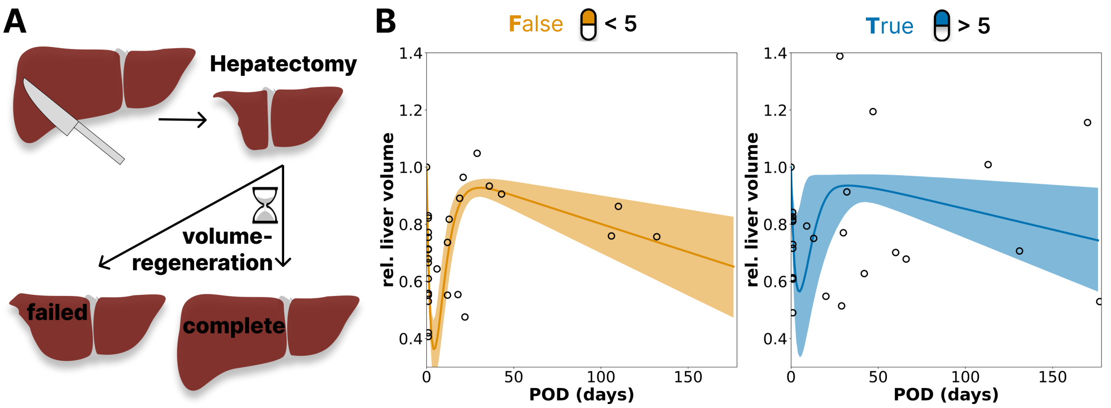

Fig. S 1. BayModTS analysis applied to postoperative liver volumes of patients subject to extended liver resection. Comparison of non-polypharmaceutic (orange) and polypharmaceutic (blue) regeneration courses after liver resection. A. Volume regeneration of human livers was monitored pre- and post-hepatectomy. B. Non-polypharmaceutic (left) and polypharmaceutic (right) liver regeneration data (o), shown along with inferred median (dark lines) and 95% Credibility Interval (CI) tubes (shaded areas).

In humans, benign and malignant liver lesions may require extensive partial resection of the liver. Clinical Computed Tomography (CT)-based liver volumetry studies can assess postoperative liver regeneration after resection. In most cases, the liver regenerates and regains its original volume within a few weeks after resection (Fig. 1A). However, various factors, such as the extent of the resection, pre-existing liver cell damage, previous illnesses, or postoperative complications influence the course of regeneration after the operation. Given the increasing number of elderly patients requiring such a resection (Reddy *et al.*, 2011), the superordinate ZeLeR-study (ethical vote: 2018-1246-Material) aims to investigate the influence of age and secondary confounding variables such as polypharmacy (= more than five medications per day). Postoperative volume regeneration of the liver was investigated in 24 patients from two different age groups (<46 years and >64 years). All patients underwent extended partial liver resection at Jena University Hospital between 03/2019 and 10/2022 as part of the study. In addition to age, influencing factors such as polypharmacy, multimorbidity, gender and extent of resection were recorded. The patients underwent preoperative imaging, usually CT and, less frequently, Magnetic Resonance Imaging (MRI). All images obtained within 6 months postoperatively were used to assess postoperative volume recovery. Liver volumes were determined using the Synapse3D software (Fujifilm).

Our aim for this study was to find differences in the regeneration courses between non-polypharmaceutical patients (False) and polypharmaceutical patients (True) (Fig. 1B). Our focus is on liver regeneration, which is relative to the preoperative liver volume. Therefore, the data are normalised to preoperative liver volume to account for differences in baseline liver volumes. It is noted that data points at later times likely correspond to liver volumes of patients with complications in the postoperative course, therefore remaining under observation. Patients without complications dropped out of the study within a few days after resection. Furthermore, in some cases, the liver volume increases rapidly after hepatectomy and temporarily exceeds the original volume, leading to values above 1.

We use BayModTS with RTF as a flexible phenomenological model to describe the resection data. Our approach captures the data of non-polypharmaceutical patients (orange curve) well (Fig. 1B left). The volume courses increase within a few weeks after resection but drop considerably afterwards. As explained before, this drop is probably an artefact of screening only patients with complications at that time. The data of polypharmaceutical patients has greater variability, which is not as well captured by the BayModTS Credibility Interval (CI) tubes (Fig. 1B right). In contrast to the non-polypharmaceutical patients, the volume decrease of the polypharmaceutical group is less pronounced on the longer time scale. The uncertainty after about 50 PODs seems not well captured by the posterior parameter ensemble prediction. The imbalance of observation times of the dataset, with most data points present before 30 PODs, might be the reason for this underestimated uncertainty, as these data points mainly determine the predictions. Further, the relative liver volume data is split between high and low values after 50 POD. A standard likelihood with squared distances (Equ. (5) main paper) can lead to a regression towards the centre when the observed data is split between high and low values. Given the limited observations after 50 POD, the regression towards the centre in the polypharmaceutic data appears to be insufficiently conservative. Therefore, more data has to be collected for this example before sound predictions can be made. Overall, interpreting these results and comparing the two groups is difficult due to the small sample size and the many confounding factors that could be addressed with a multi-factorial analysis.

In summary, BayModTS improved the visualisation of this heterogeneous liver volume dataset from hepatectomy patients. We would like to point out that the results for challenging data sets like this should always be interpreted cautiously. Here, the posterior parameter ensemble prediction shows that the data on polypharmaceutical patients are not yet adequately captured by the RTF and that a better data basis for predictions is needed. We plan to extend this analysis as more data becomes available. Particularly in patients with pre-existing diseases, knowledge of the progression of liver regeneration could improve the planning and execution of resections.

Pharmacokinetic model for drug metabolisation in steatotic mice

To validate the RTF predictions and demonstrate BayModTS's easy applicability to general ODE models, we applied a pharmacokinetic (PK) ODE model to the drug metabolisation data of steatotic mice (second application example). Our PK model is a two-compartment model with a central and a peripheral compartment (Fig. 2B). The drug is injected intraperitoneally in

the gut compartment and subsequently absorbed by the central compartment via the absorption rate $kabs$. The distribution between the central compartment and the peripheral compartment is described by the distribution parameter Q, and the drug clearance from the central compartment is described by the clearance rate CL (Equ. (S 2), (S 3)). The concentration in the central compartment corresponds to our observed data. Drug injection is modelled via an application curve to the gut compartment that mimics the experimental injection speed of 200µl per 30 seconds (Equ. (S 1)). The injection curve's integral equals the injection amount in the injection period.

The predicted medians and CI tubes for caffeine and codeine were similar for the RTF and PK approaches (Figure 2C, D). The main difference was the smaller CIs of the PK models at 6h. The PK model structure can explain the smaller CI tubes at 6h in the pharmacokinetic model, preventing a second concentration increase without renewed drug injection. Further, the PK model showed a rapid midazolam appearance, compared to a smoother midazolam appearance in the RTF model. Overall, comparing the RTF to PK BayModTS results shows that the RTF predictions and uncertainties were meaningful. The conclusions drawn from the analysis are identical for both approaches: (i) macrovesicular periportal steatosis (4 weeks) seems to delay the clearance of all test drugs, and (ii) peak concentrations of caffeine and midazolam were higher in macrovesicular steatosis.

The PK model further allows us to interpret differences on the parameter level. For example, the clearance rate (CL) was delayed in macrosteatotic mice for all three drugs (Fig. 3 first column). For the absorption rate, both steatotic conditions seem to increase the absorption rate for all drugs (Fig. 3 second column). The distribution coefficient Q was not identifiable, hampering a comparison between conditions (Fig. 3 third column). More data would be needed to improve practical identifiability.

In short, the PK and RTD's BayModTS results agree. Using the PK models in the BayModTS workflow demonstrates a smooth transition from predictive Bayesian analysis to mechanistic computational modelling.

$$x_{gut} = \frac{166.989 \cdot 5 \cdot 0.029}{M_{drug}} \cdot (1 - e^{\frac{-t}{\tau}}) \cdot e^{\frac{-t}{\tau}} \tag{S 1}$$

$$\frac{dx_{cent}}{dt} = kabs \cdot x_{gut} + Q \cdot (x_{peri} - x_{cent}) - CL \cdot x_{cent} \tag{S 2}$$

$$\frac{dx_{peri}}{dt} = Q \cdot (x_{cent} - x_{peri}) \tag{S 3}$$

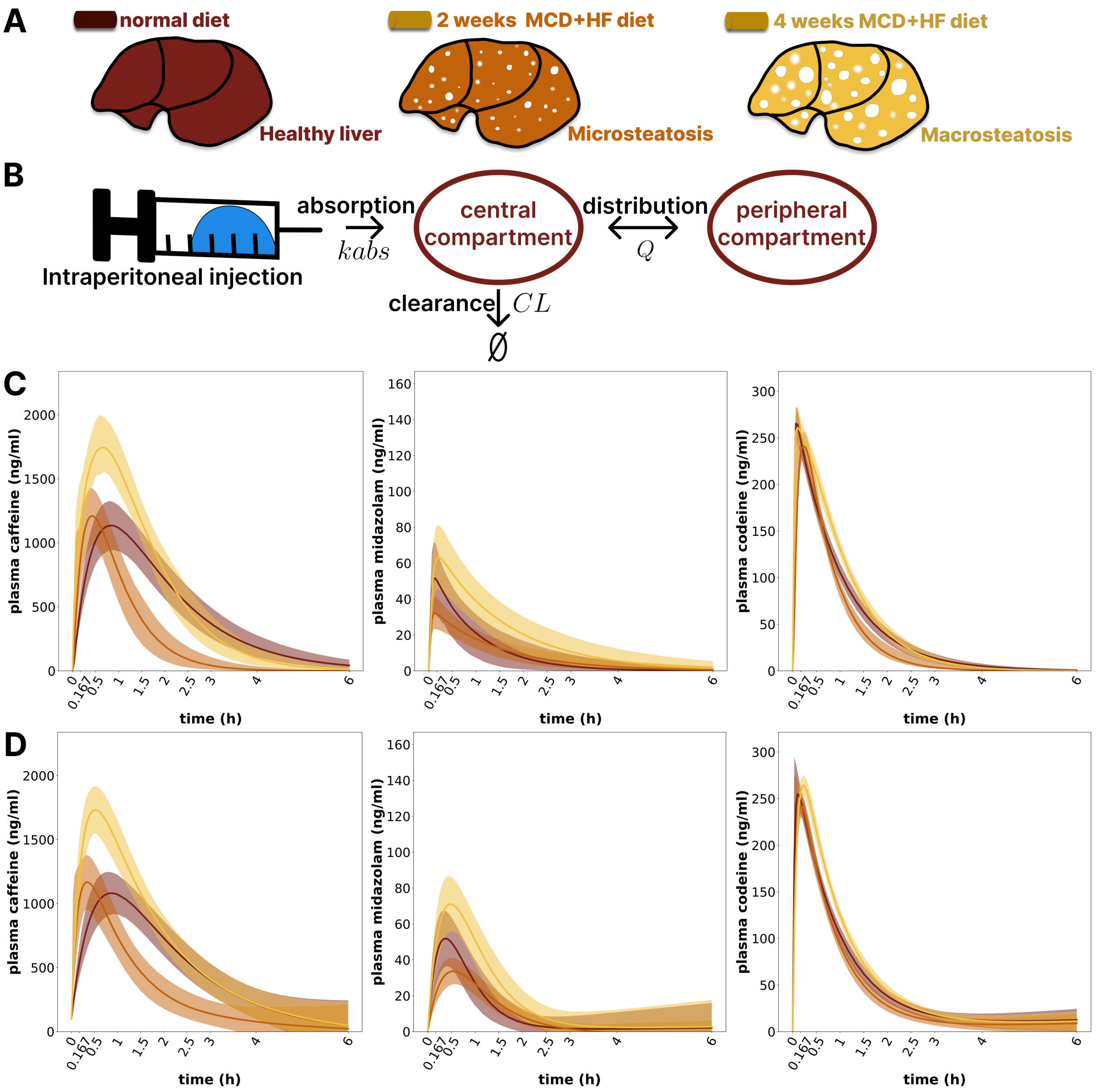

Fig. S 2. BayModTS analysis with pharmacokinetic model validates Retarded Transient Function (RTF) results on drug metabolisation in steatotic mice. A. Mice were fed 2 weeks with a normal diet and with Methionine-Choline Deficient (MCD) and High Fat (HF) diet for either 2 or 4 weeks. The MCD+HF diet induces hepatic microsteatosis after 2 weeks and macrosteatosis after 4 weeks. B. Illustration of the pharmacokinetic model. Caffeine (5 mg/kg), midazolam (2 mg/kg), and codeine (2 mg/kg) were injected intraperitoneally, absorbed by the central compartment and distributed to a peripheral compartment or cleared. C. Median ensemble prediction (dark lines) and 95% CI tubes of the BayModTS analysis of the pharmacokinetic model. D. Median ensemble prediction (dark lines) and 95% CI tubes of the BayModTS analysis with RTFs from Fig. 3.

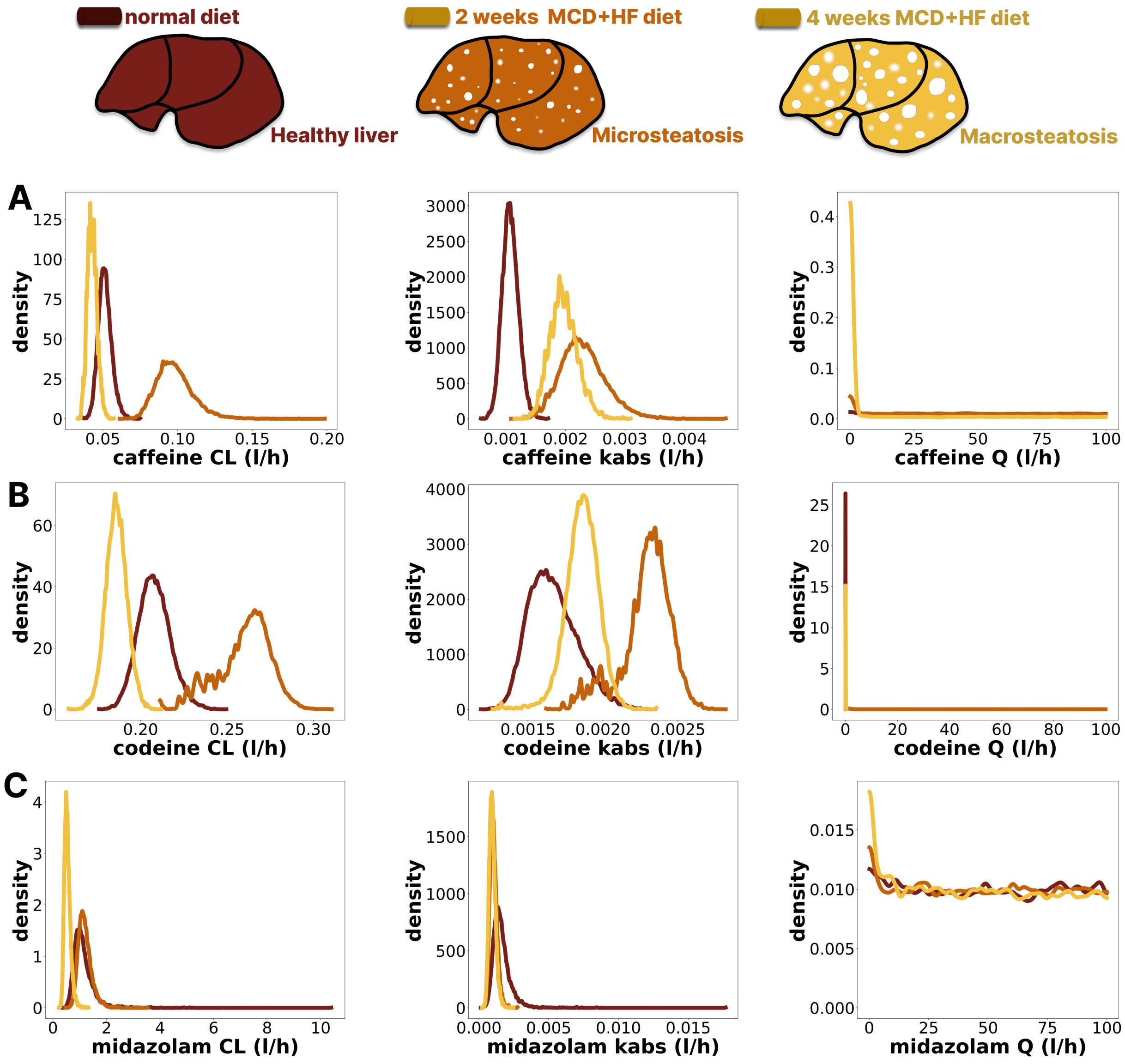

Fig. S 3. Kernel density estimates of marginal pharmacokinetic parameters. Kernel Density Estimates (KDE) of the clearance rate CL, the absorption rate $kabs$ and the distribution coefficient Q for caffeine (A), midazolam (B), and codeine (C). Every 10th parameter value of the converged chain was used to calculate the KDE. The Effective Sample Size (ESS) was 473410 for caffeine control, 473799 for caffeine 2 weeks, 467572 for caffeine 4 weeks, 323562 for midazolam control, 465032 for midazolam 2 weeks, 471333 for midazolam 4 weeks, 473337 for codeine control, 377339 for codeine 2 weeks, 471257 for codeine 4 weeks.

References

Kruschke, J. K. (2021). Bayesian Analysis Reporting Guidelines. *Nature Human Behaviour*, **5**(10), 1282–1291.

Reddy, S. K. *et al.* (2011). Major liver resection in elderly patients: a multi-institutional analysis. *J Am Coll Surg*, **212**(5), 787–95.

Sampling scatters

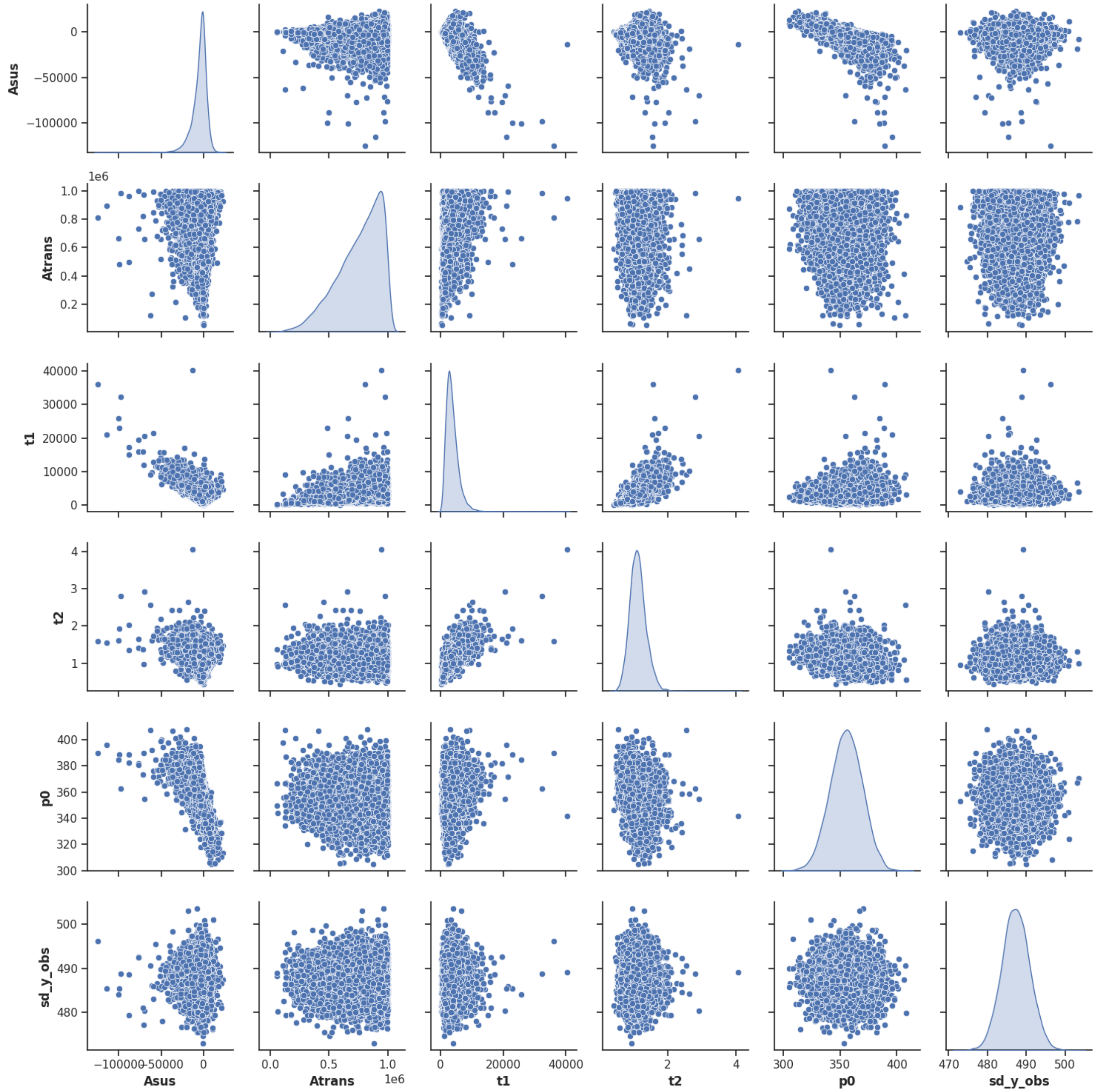

Fig. S 4. Posterior scatter plots of RTF model parameters for the RML in application example 1. 1D marginals and 2D parameter scatter plots from MCMC sampling of the posterior distribution for the RML. An immediate response ($T_{shift} = -2$) and identical response times ($t1 = t11$) were assumed. T_{range} was set to the range of the experimental data ($T_{range} = 5$). The Effective Sample Size (ESS) was 9153.3 samples. According to the Bayesian Analysis Reporting Guidelines (Kruschke, 2021), results were validated with a broader prior. Results with the broader prior can be found on DaRUS.

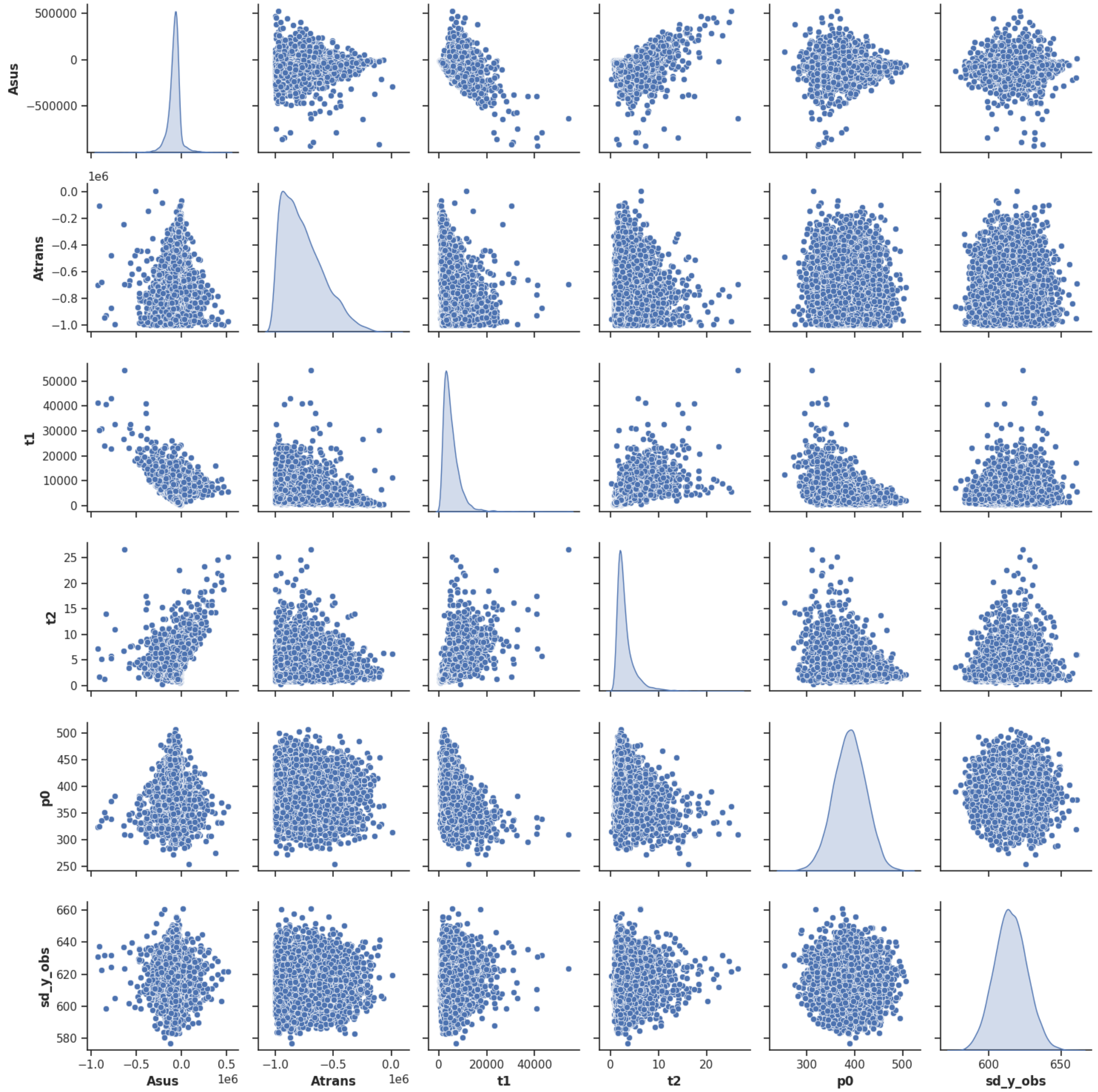

Fig. S 5. Posterior scatter plots of RTF model parameters for the LML in application example 1. 1D marginals and 2D parameter scatter plots from MCMC sampling of the posterior distribution for the LML. An immediate response ($T_{shift} = -2$) and identical response times ($t1 = t11$) were assumed. T_{range} was set to the range of the experimental data ($T_{range} = 5$). The Effective Sample Size (ESS) was 2812.5 samples. According to the Bayesian Analysis Reporting Guidelines (Kruschke, 2021), results were validated with a broader prior. Results with the broader prior can be found on DaRUS.

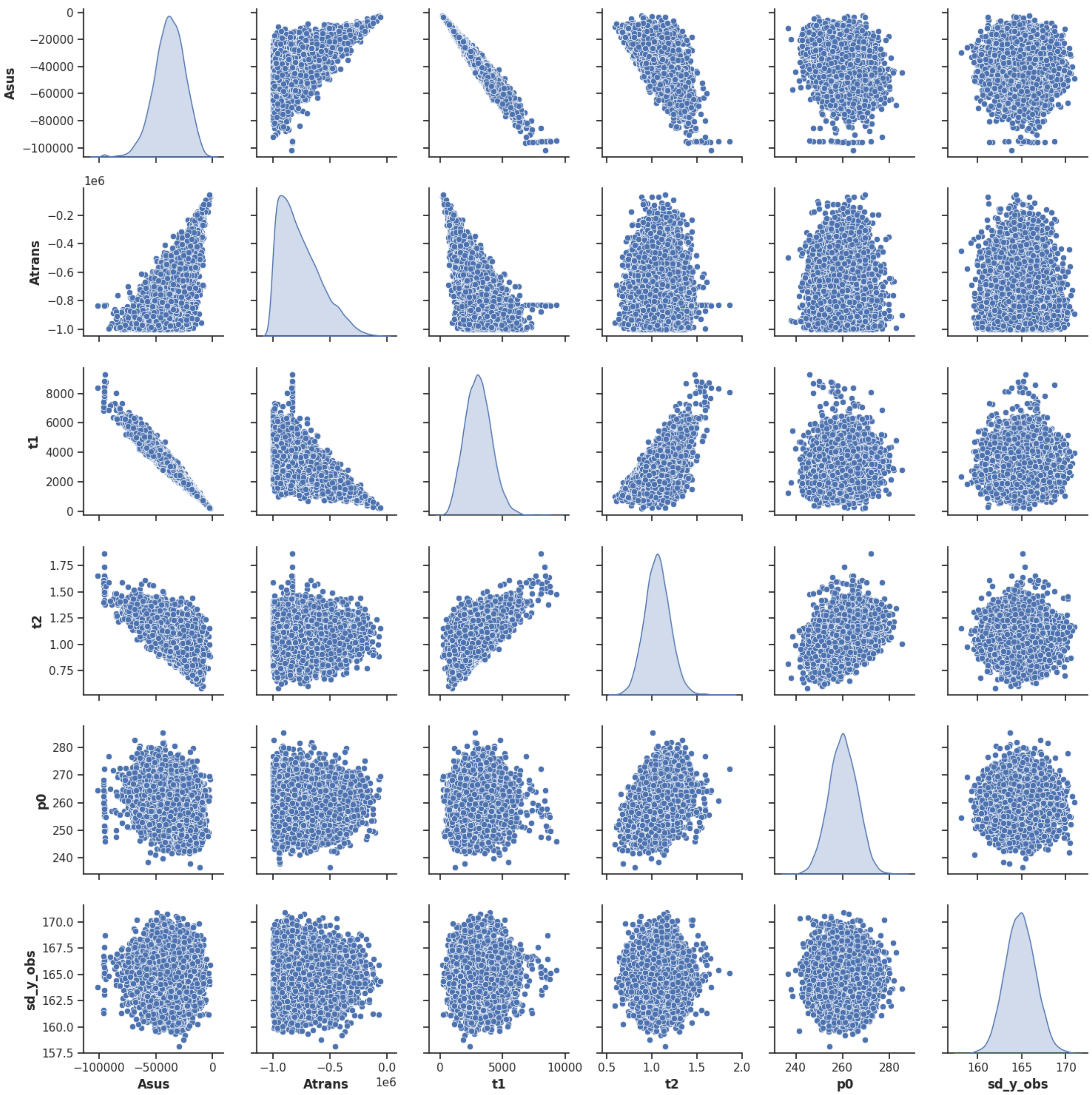

Fig. S 6. Posterior scatter plots of RTF model parameters for the RL in application example 1. 1D marginals and 2D parameter scatter plots from MCMC sampling of the posterior distribution for the RL. An immediate response ($T_{shift} = -2$) and identical response times ($t1 = t11$) were assumed. T_{range} was set to the range of the experimental data ($T_{range} = 5$). The Effective Sample Size (ESS) was 16600.4 samples. According to the Bayesian Analysis Reporting Guidelines (Kruschke, 2021), results were validated with a broader prior. Results with the broader prior can be found on DaRUS.

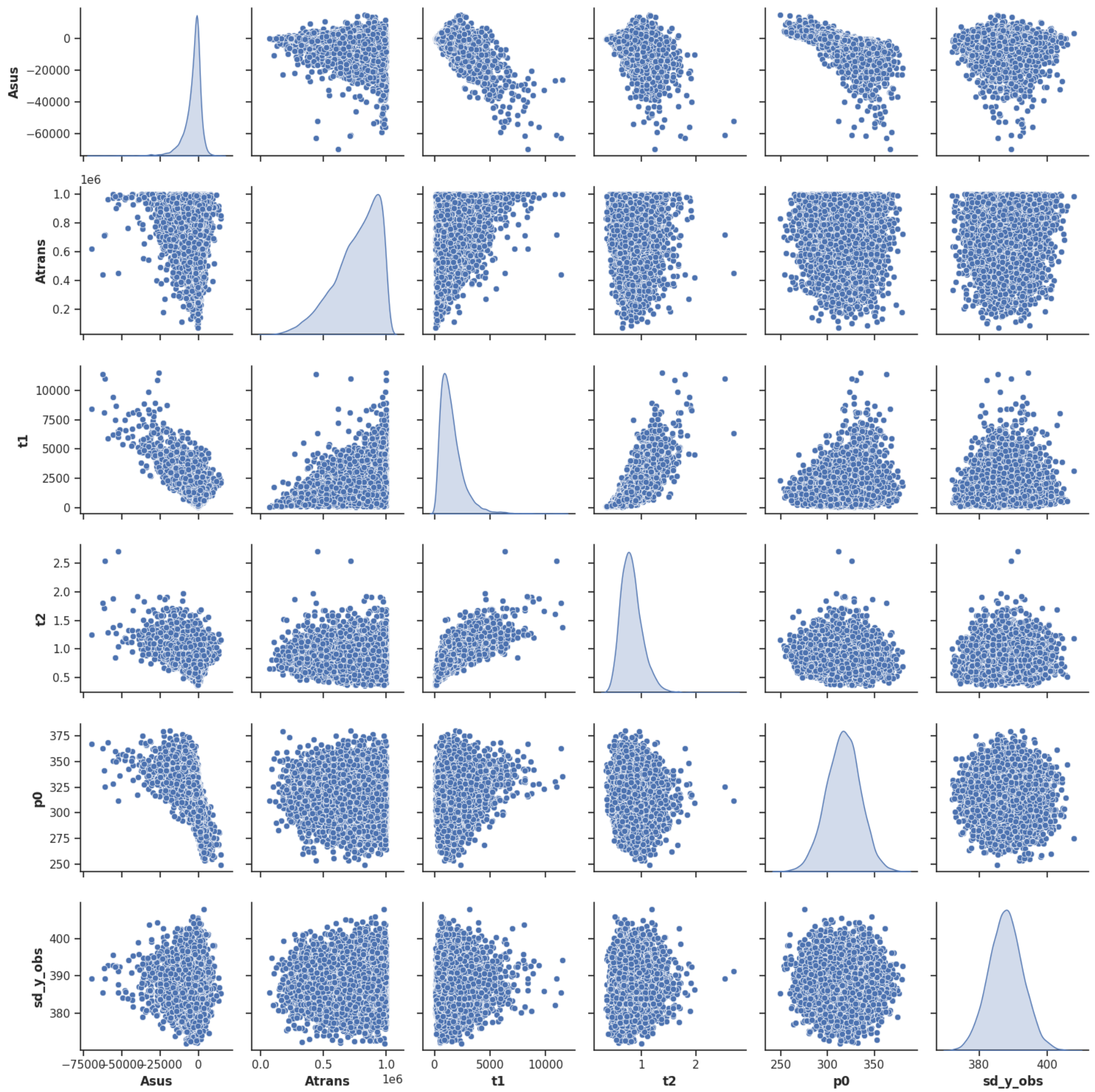

Fig. S 7. Posterior scatter plots of RTF model parameters for the CL in application example 1. 1D marginals and 2D parameter scatter plots from MCMC sampling of the posterior distribution for the CL. An immediate response ($T_{shift} = -2$) and identical response times ($t1 = t11$) were assumed. T_{range} was set to the range of the experimental data ($T_{range} = 5$). The Effective Sample Size (ESS) was 1216 samples. According to the Bayesian Analysis Reporting Guidelines (Kruschke, 2021), results were validated with a broader prior. Results with the broader prior can be found on DaRUS.

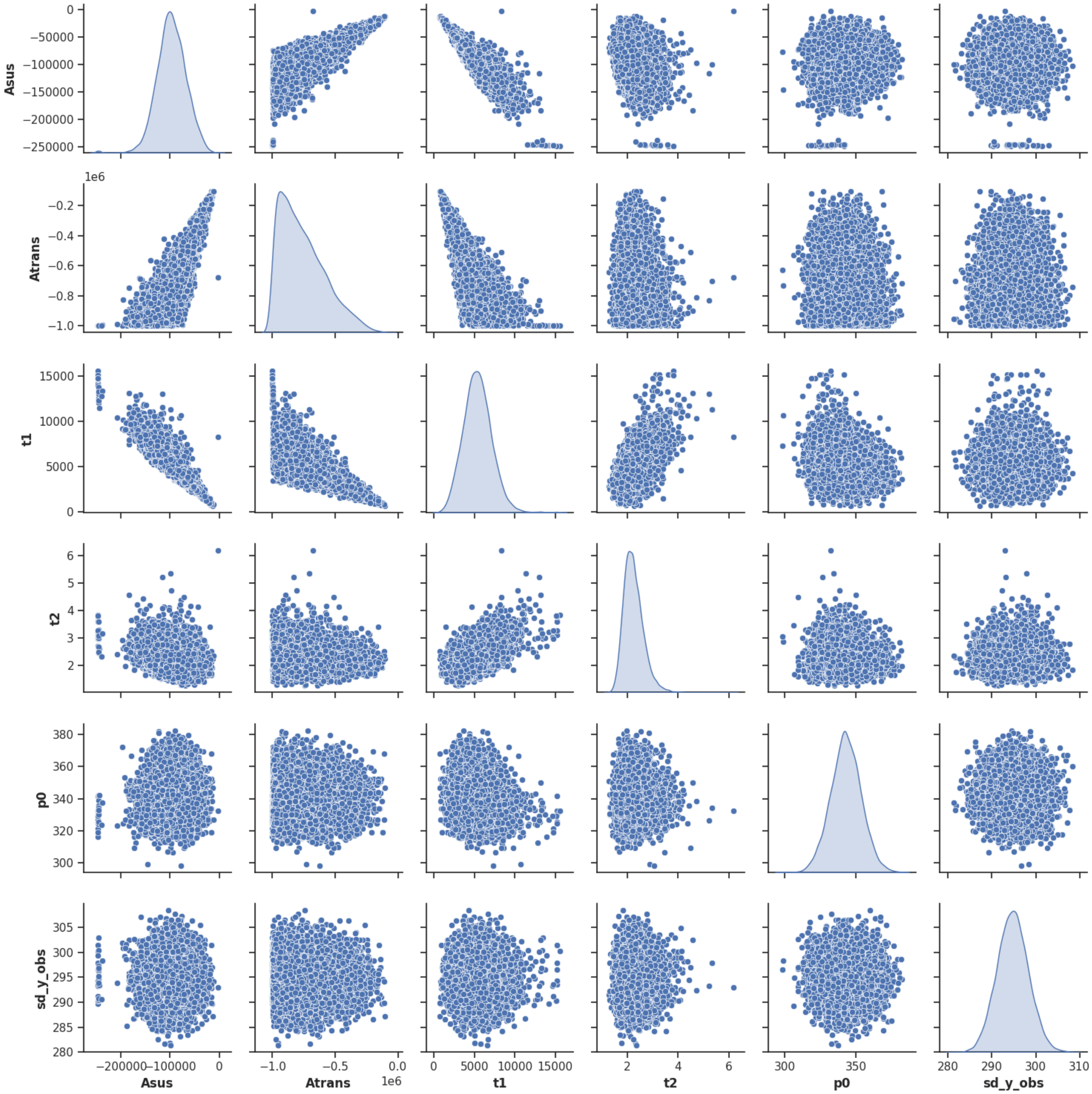

Fig. S 8. Posterior scatter plots of RTF model parameters for the LLL in application example 1. 1D marginals and 2D parameter scatter plots from MCMC sampling of the posterior distribution for the LLL. An immediate response ($T_{shift} = -2$) and identical response times ($t1 = t11$) were assumed. T_{range} was set to the range of the experimental data ($T_{range} = 5$). The Effective Sample Size (ESS) was 15102.2 samples. According to the Bayesian Analysis Reporting Guidelines (Kruschke, 2021), results were validated with a broader prior. Results with the broader prior can be found on DaRUS.

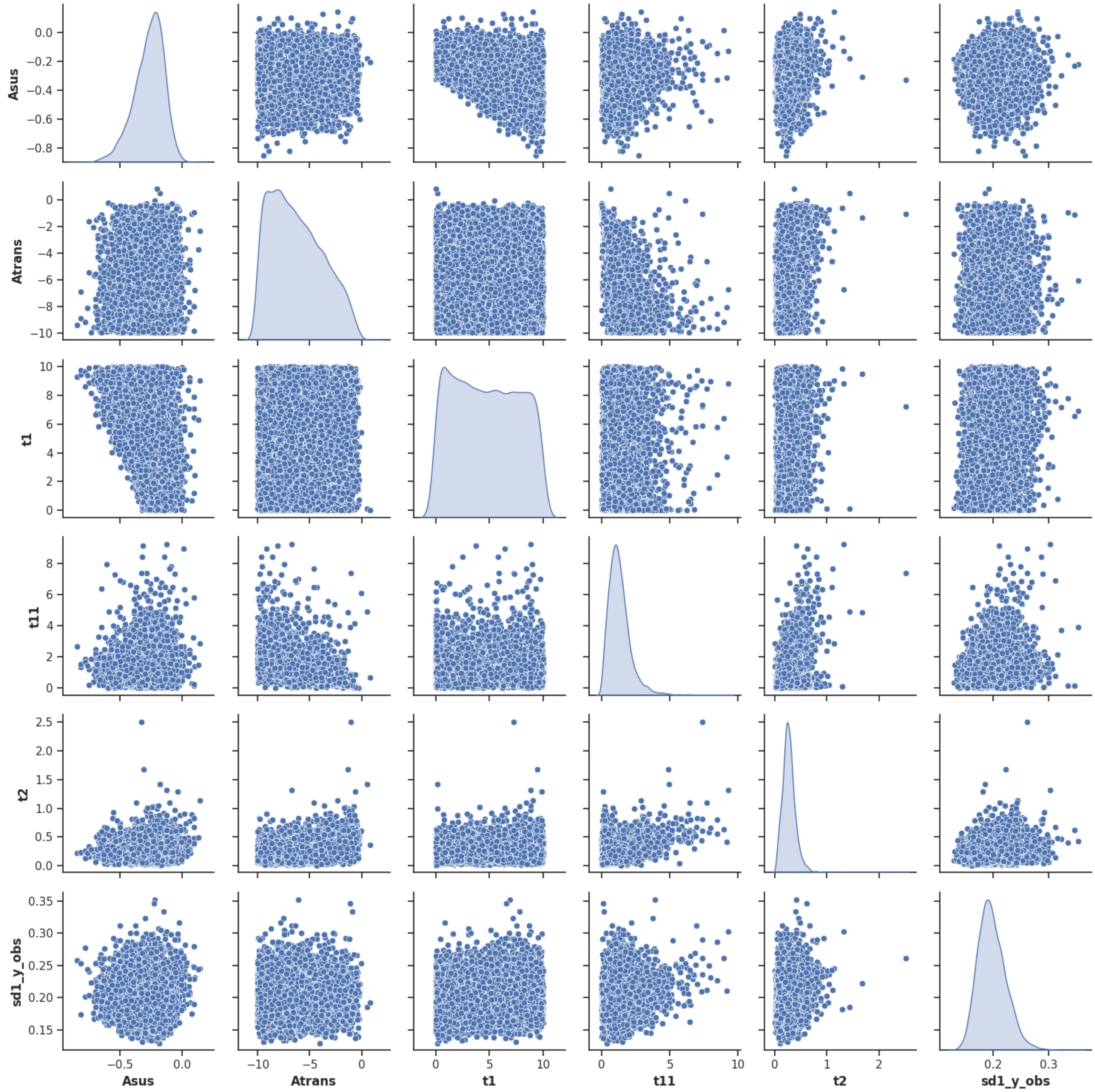

Fig. S 9. Posterior scatter plots of RTF model parameters for the polypharmaceutic group in application example 2. 1D marginals and 2D parameter scatter plots from MCMC sampling of the posterior distribution for the polypharmaceutic group. An immediate response ($T_{shift} = -2$) was assumed, and the initial liver volume $p0$ was normalised to 1 for each patient. T_{range} was set to the range of the experimental data ($T_{range} = 177$). The Effective Sample Size (ESS) was 34832.8 samples. According to the Bayesian Analysis Reporting Guidelines (Kruschke, 2021), results were validated with a broader prior. Results with the broader prior can be found on DaRUS.

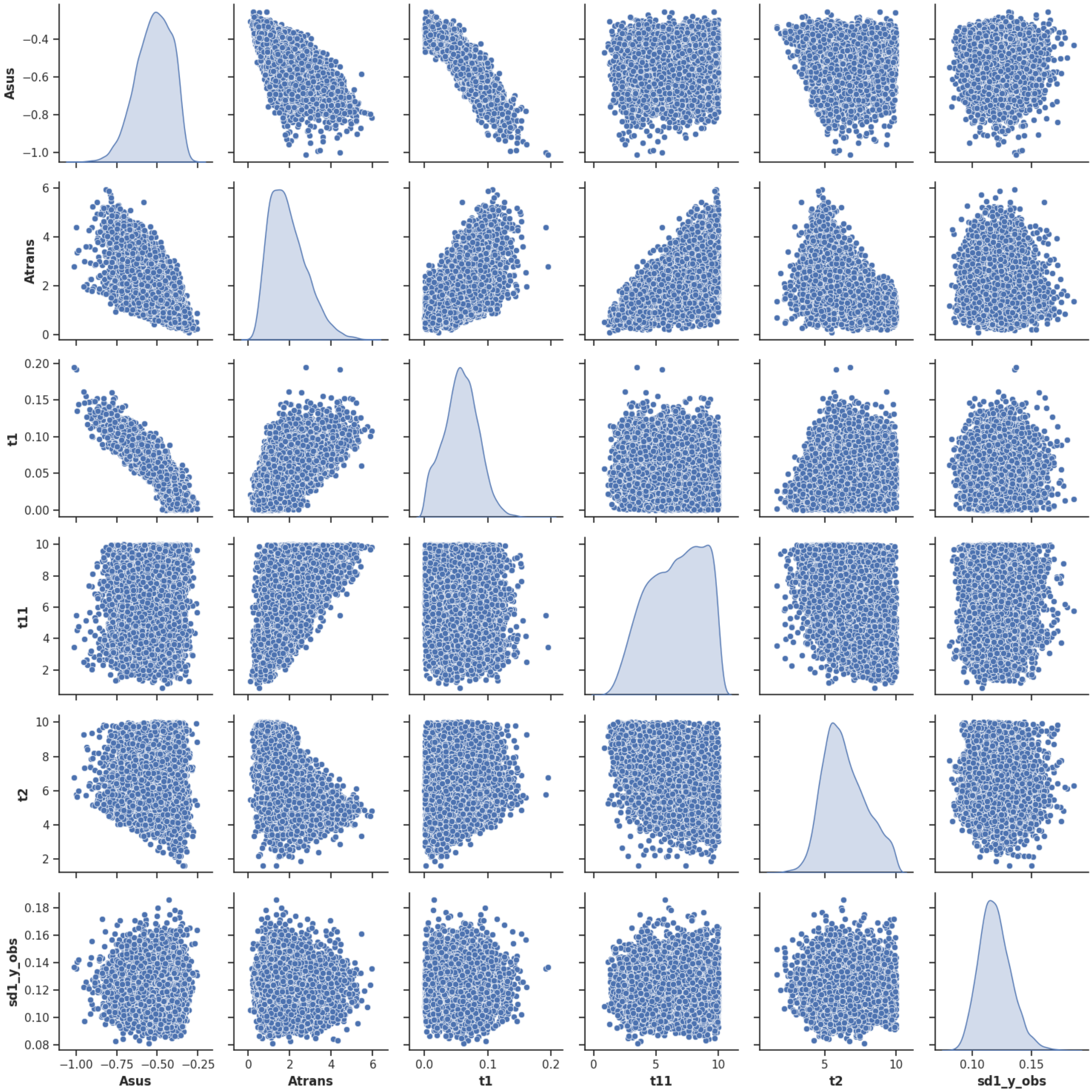

Fig. S 10. Posterior scatter plots of RTF model parameters for the non-polypharmaceutic group in application example 2. 1D marginals and 2D parameter scatter plots from MCMC sampling of the posterior distribution for the non-polypharmaceutic group. An immediate response ($T_{shift} = -2$) was assumed, and the initial liver volume $p0$ was normalised to 1 for each patient. T_{range} was set to the range of the experimental data ($T_{range} = 132$).The Effective Sample Size (ESS) was 37410.1 samples. According to the Bayesian Analysis Reporting Guidelines (Kruschke, 2021), results were validated with a broader prior. Results with the broader prior can be found on DaRUS.

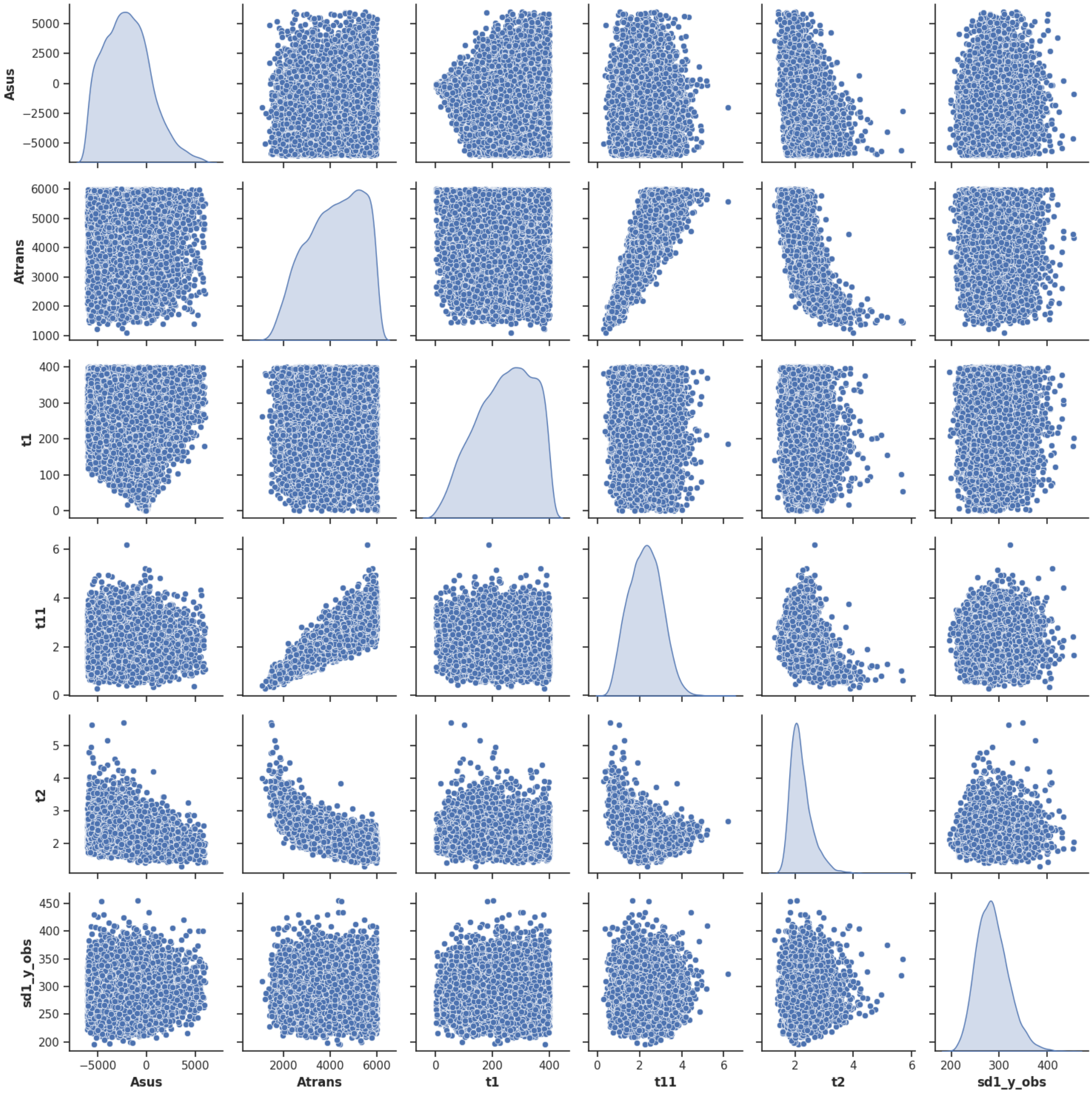

Fig. S 11. Posterior scatter plots of RTF model parameters for the caffeine control group in application example 3. 1D marginals and 2D parameter scatter plots from MCMC sampling of the posterior distribution for the caffeine control group. An immediate response ($T_{shift} = -2$) was assumed, and the initial drug concentration before injection was set to zero ($p_0 = 0$). T_{range} was set to the range of the experimental data ($T_{range} = 6$). The Effective Sample Size (ESS) was 15932.5 samples. According to the Bayesian Analysis Reporting Guidelines (Kruschke, 2021), results were validated with a broader prior. Results with the broader prior can be found on DaRUS.

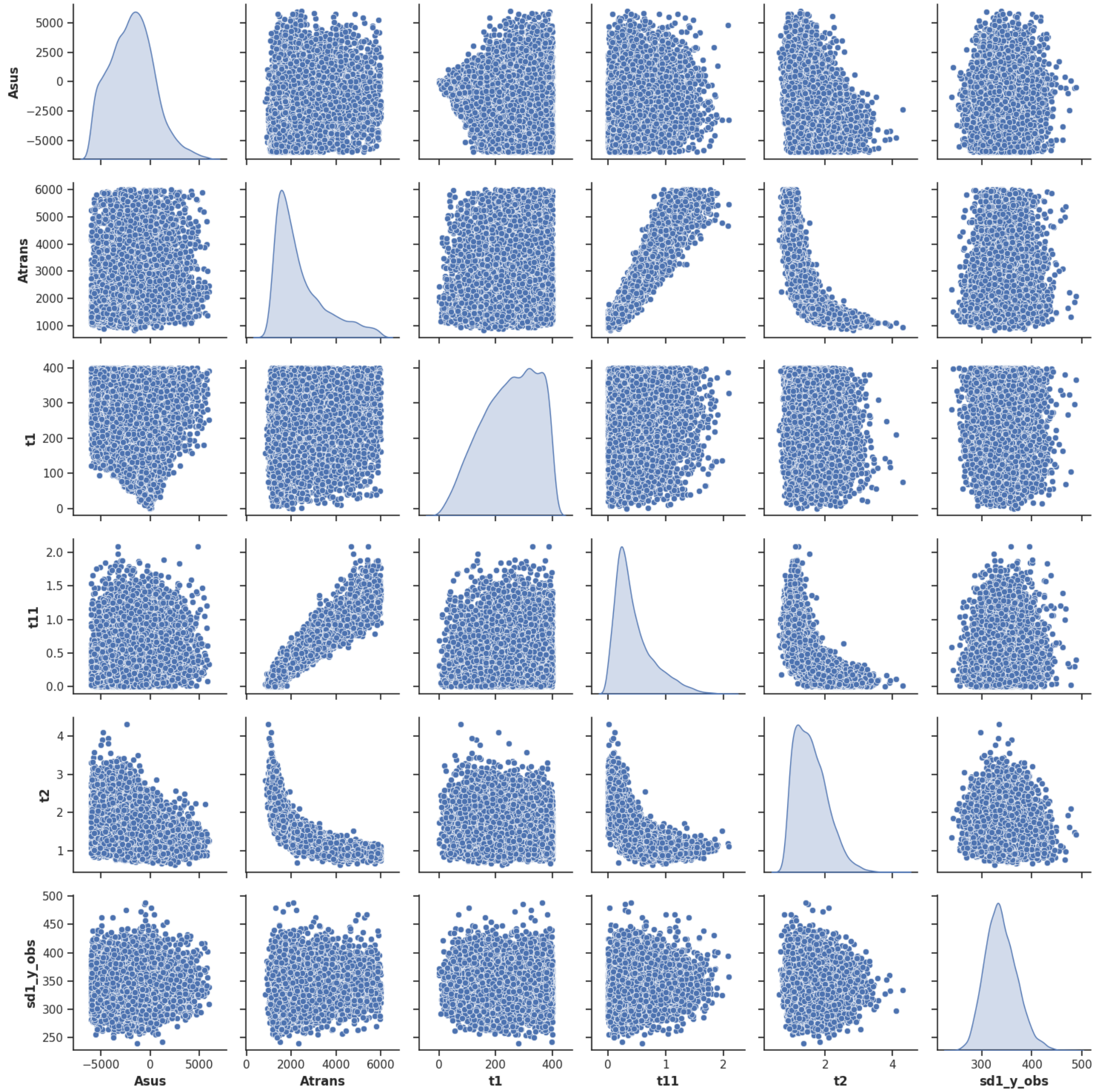

Fig. S 12. Posterior scatter plots of RTF model parameters for the caffeine 2 weeks group in application example 3. 1D marginals and 2D parameter scatter plots from MCMC sampling of the posterior distribution for the caffeine 2 weeks group. An immediate response ($T_{shift} = -2$) was assumed, and the initial drug concentration before injection was set to zero ($p_0 = 0$). T_{range} was set to the range of the experimental data ($T_{range} = 6$). The Effective Sample Size (ESS) was 5841.1 samples. According to the Bayesian Analysis Reporting Guidelines (Kruschke, 2021), results were validated with a broader prior. Results with the broader prior can be found on DaRUS.

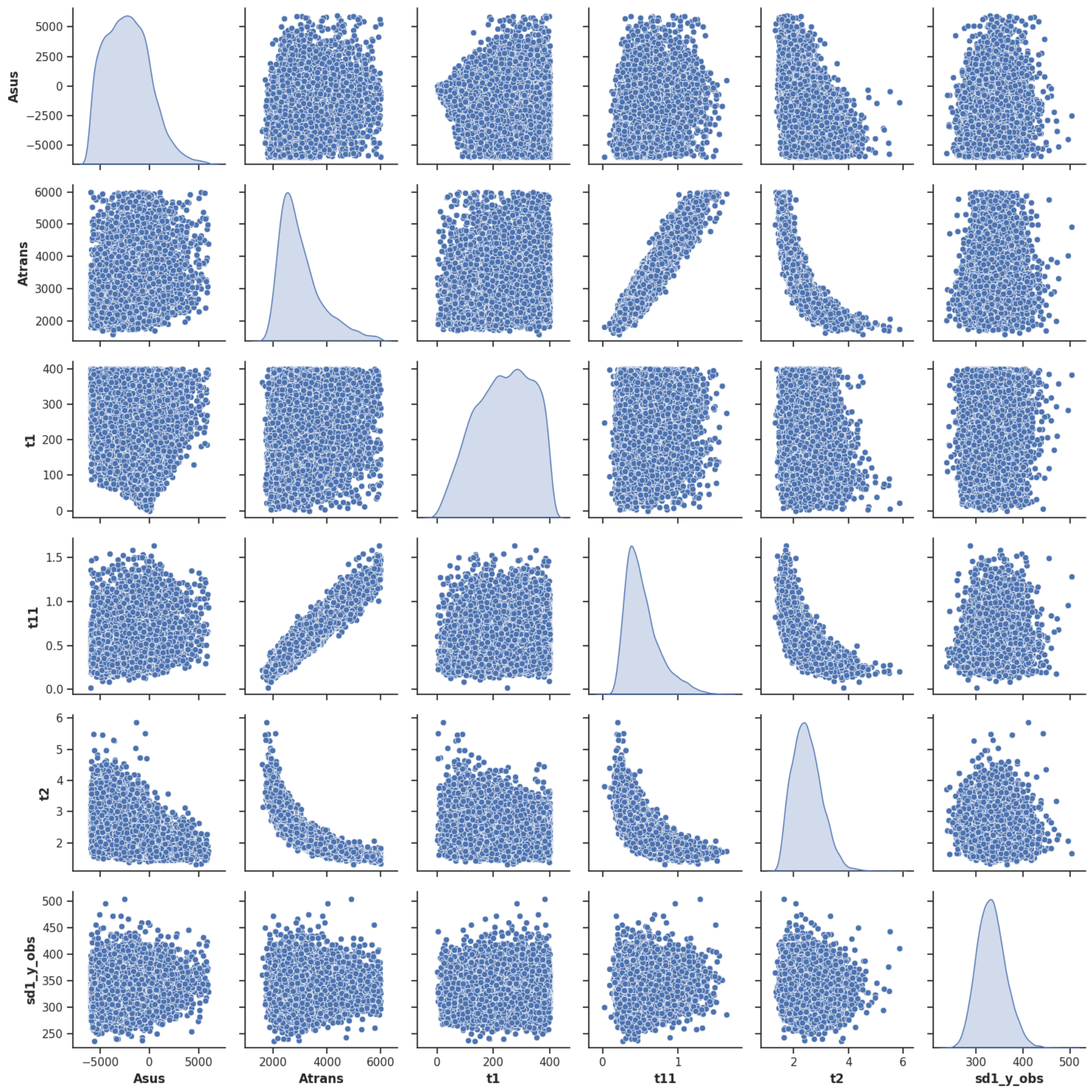

Fig. S 13. Posterior scatter plots of RTF model parameters for the caffeine 4 weeks group in application example 3. 1D marginals and 2D parameter scatter plots from MCMC sampling of the posterior distribution for the caffeine 4 weeks group. An immediate response ($T_{shift} = -2$) was assumed, and the initial drug concentration before injection was set to zero ($p_0 = 0$). T_{range} was set to the range of the experimental data ($T_{range} = 6$). The Effective Sample Size (ESS) was 10574.2 samples. According to the Bayesian Analysis Reporting Guidelines (Kruschke, 2021), results were validated with a broader prior. Results with the broader prior can be found on DaRUS.

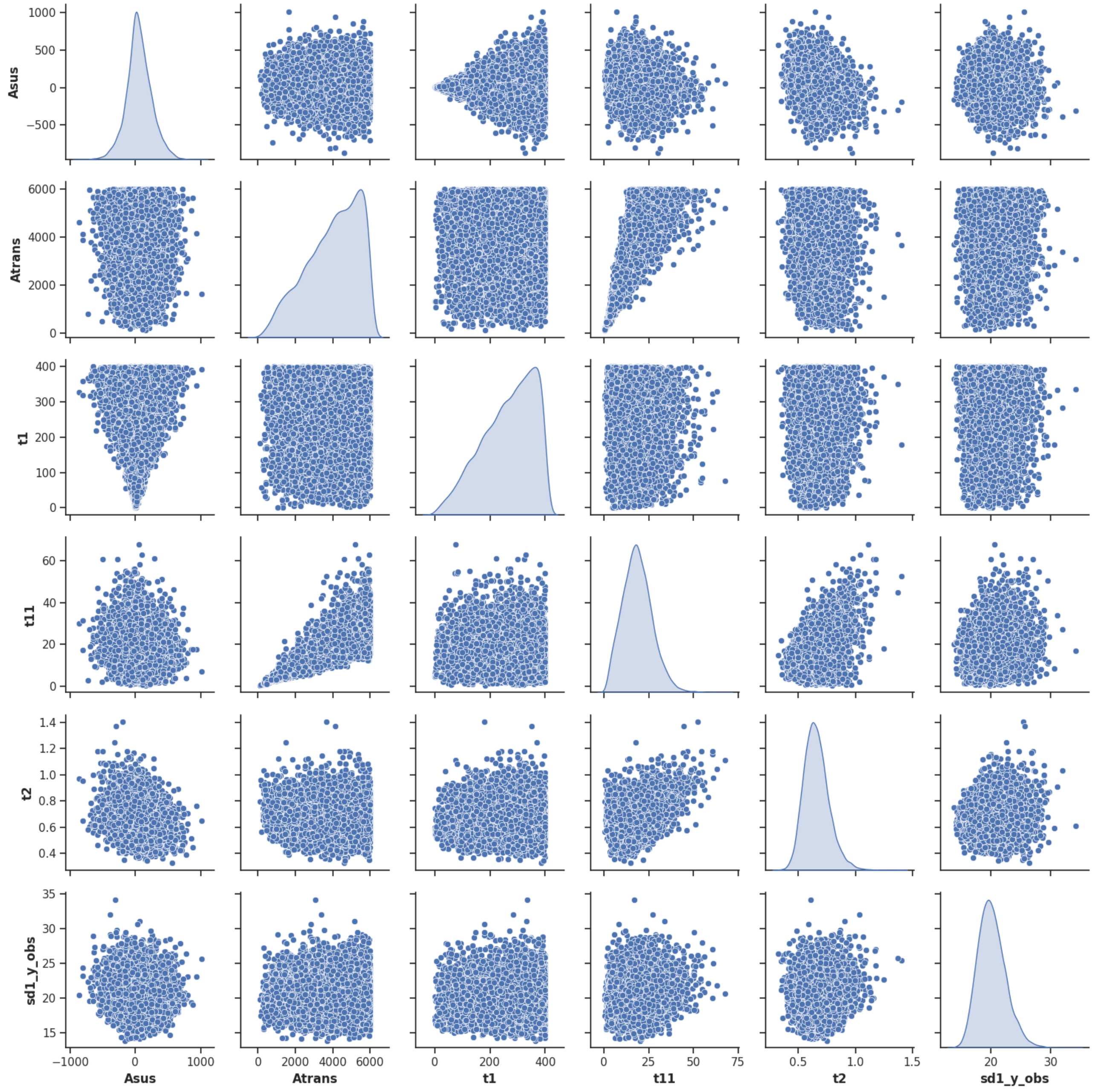

Fig. S 14. Posterior scatter plots of RTF model parameters for the midazolam control group in application example 3. 1D marginals and 2D parameter scatter plots from MCMC sampling of the posterior distribution for the midazolam control group. An immediate response ($T_{shift} = -2$) was assumed, and the initial drug concentration before injection was set to zero ($p_0 = 0$). T_{range} was set to the range of the experimental data ($T_{range} = 6$). The Effective Sample Size (ESS) was 23464.8 samples. According to the Bayesian Analysis Reporting Guidelines (Kruschke, 2021), results were validated with a broader prior. Results with the broader prior can be found on DaRUS.

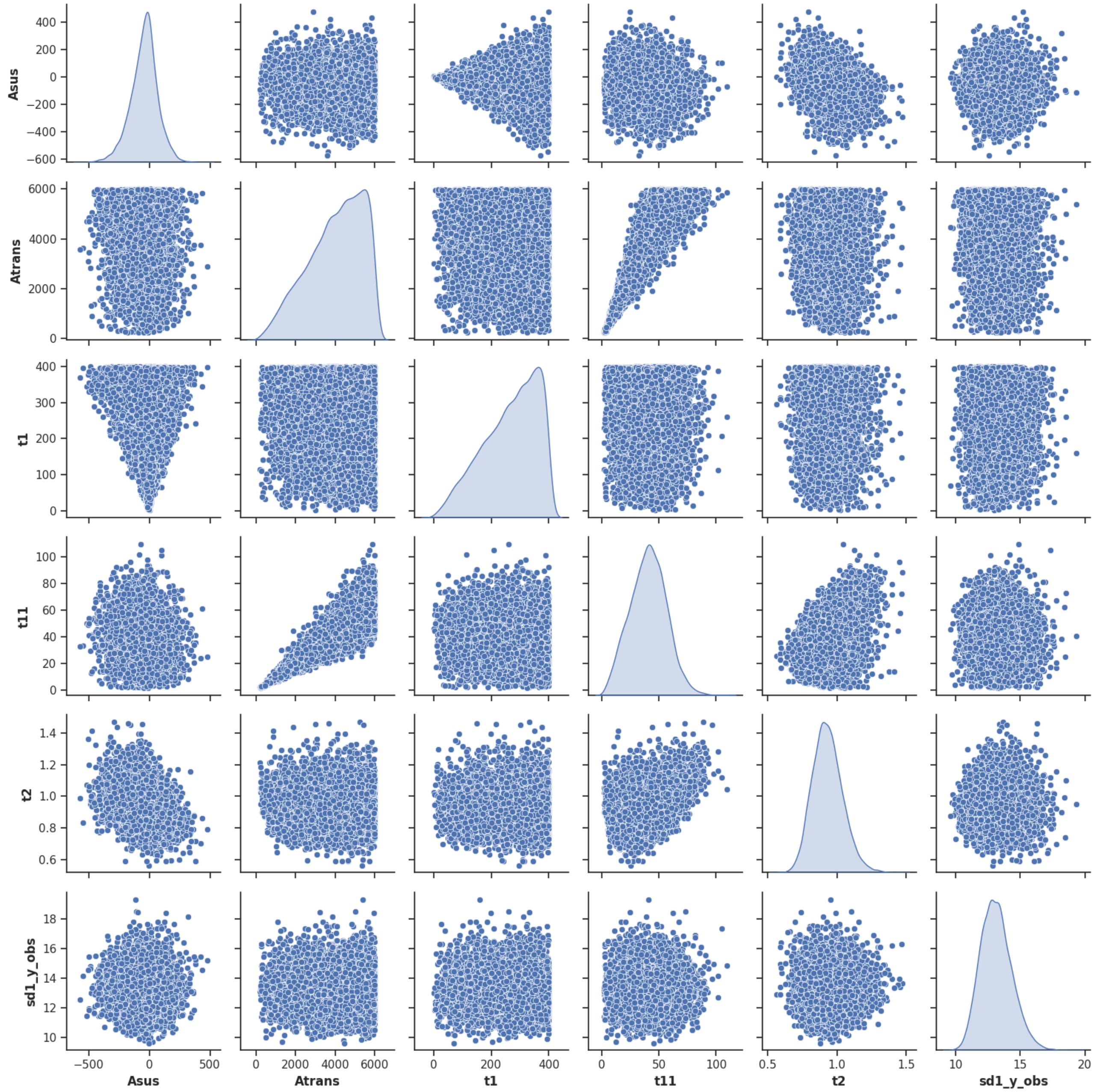

Fig. S 15. Posterior scatter plots of RTF model parameters for the midazolam 2 weeks group in application example 3. 1D marginals and 2D parameter scatter plots from MCMC sampling of the posterior distribution for the midazolam 2 weeks group. An immediate response ($T_{shift} = -2$) was assumed, and the initial drug concentration before injection was set to zero ($p_0 = 0$). T_{range} was set to the range of the experimental data ($T_{range} = 6$). The Effective Sample Size (ESS) was 27316.8 samples. According to the Bayesian Analysis Reporting Guidelines (Kruschke, 2021), results were validated with a broader prior. Results with the broader prior can be found on DaRUS.

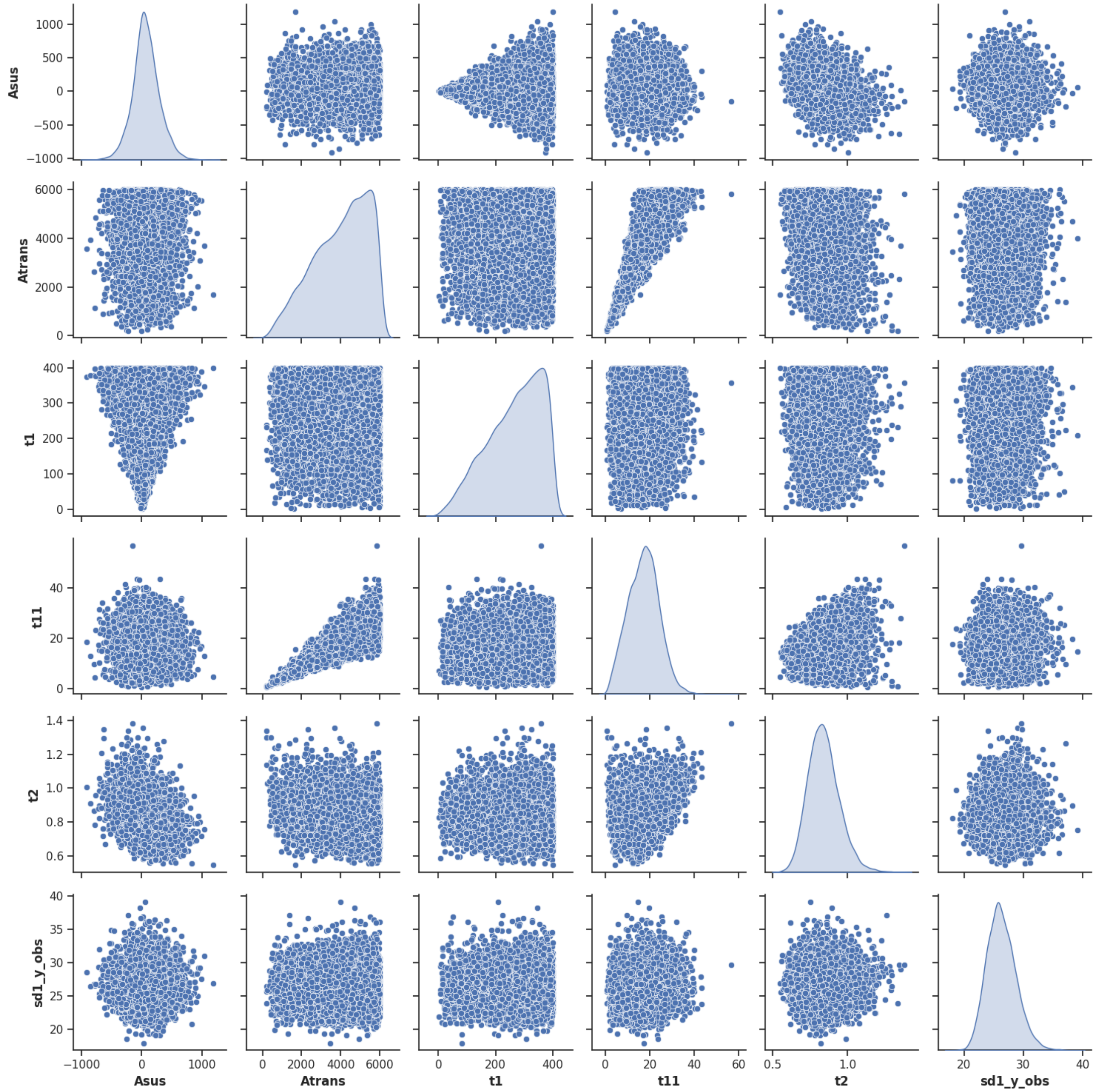

Fig. S 16. Posterior scatter plots of RTF model parameters for the midazolam 4 weeks group in application example 3. 1D marginals and 2D parameter scatter plots from MCMC sampling of the posterior distribution for the midazolam 4 weeks group. An immediate response ($T_{shift} = -2$) was assumed, and the initial drug concentration before injection was set to zero ($p_0 = 0$). T_{range} was set to the range of the experimental data ($T_{range} = 6$). The Effective Sample Size (ESS) was 23259 samples. According to the Bayesian Analysis Reporting Guidelines (Kruschke, 2021), results were validated with a broader prior. Results with the broader prior can be found on DaRUS.

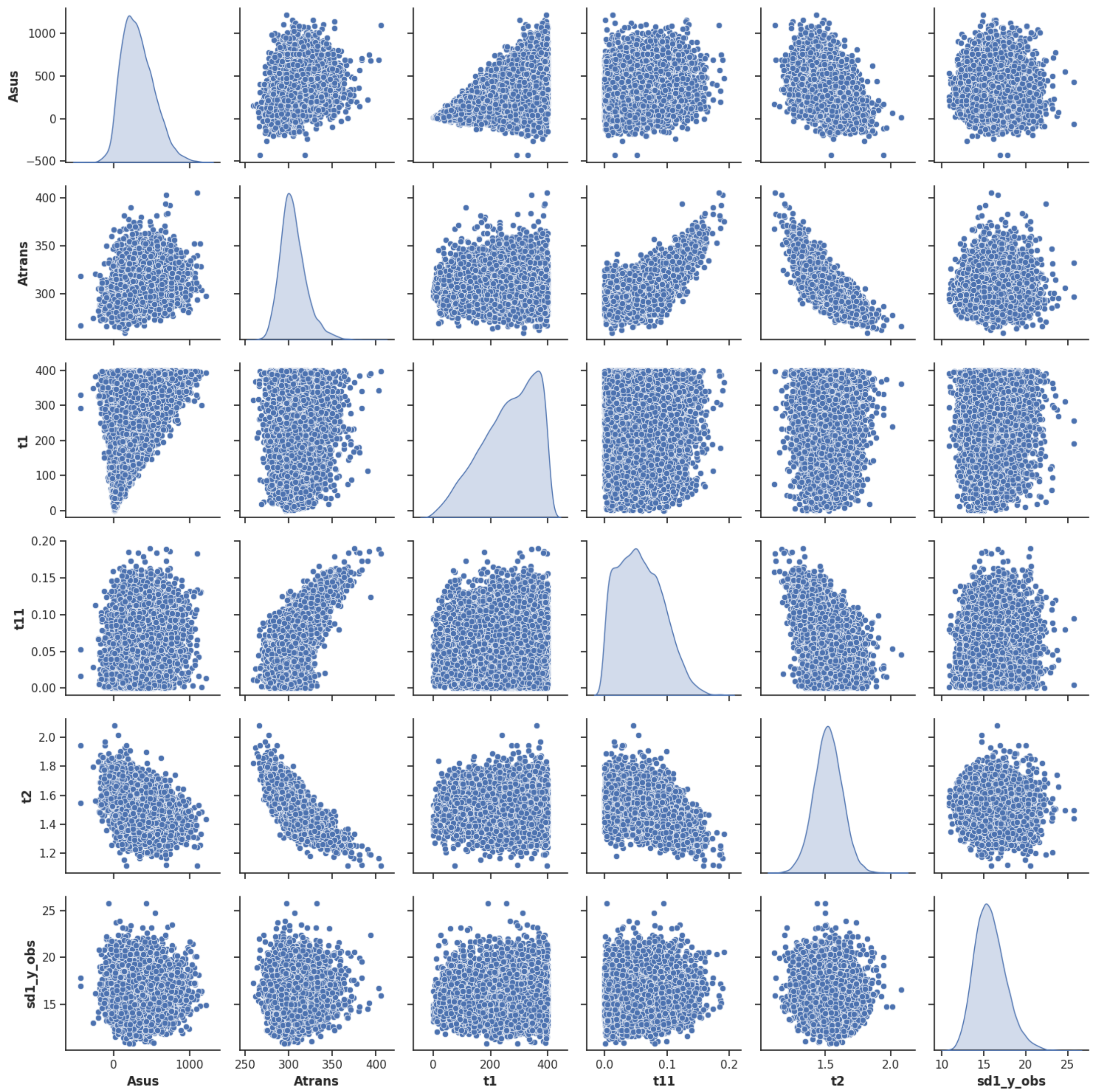

Fig. S 17. Posterior scatter plots of RTF model parameters for the codeine control group in application example 3. 1D marginals and 2D parameter scatter plots from MCMC sampling of the posterior distribution for the codeine control group. An immediate response ($T_{shift} = -2$) was assumed, and the initial drug concentration before injection was set to zero ($p_0 = 0$). T_{range} was set to the range of the experimental data ($T_{range} = 6$). The Effective Sample Size (ESS) was 29905.3 samples. According to the Bayesian Analysis Reporting Guidelines (Kruschke, 2021), results were validated with a broader prior. Results with the broader prior can be found on DaRUS.

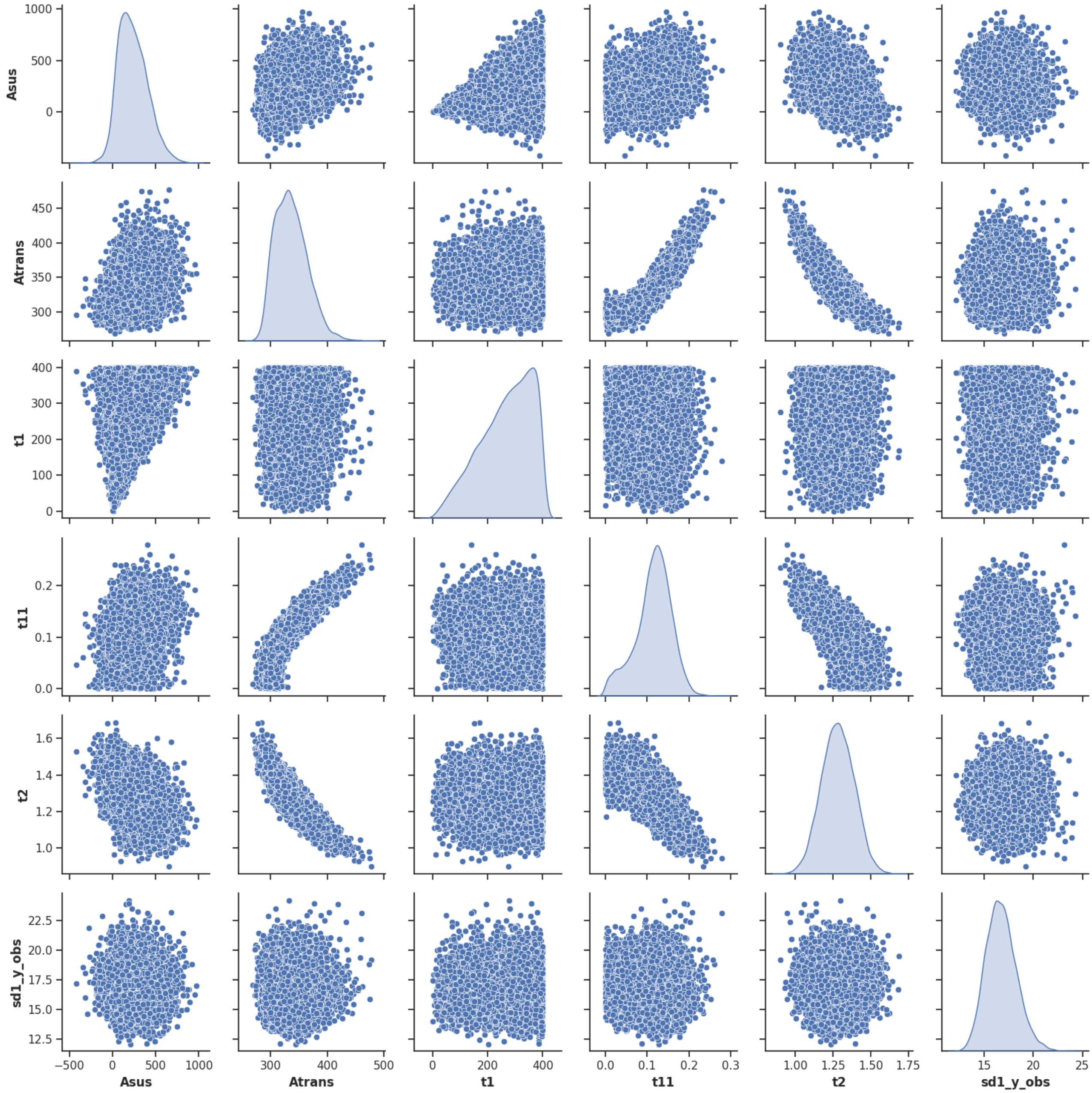

Fig. S 18. Posterior scatter plots of RTF model parameters for the codeine 2 weeks group in application example 3. 1D marginals and 2D parameter scatter plots from MCMC sampling of the posterior distribution for the codeine 2 weeks group. An immediate response ($T_{shift} = -2$) was assumed, and the initial drug concentration before injection was set to zero ($p_0 = 0$). T_{range} was set to the range of the experimental data ($T_{range} = 6$). The Effective Sample Size (ESS) was 24403 samples. According to the Bayesian Analysis Reporting Guidelines (Kruschke, 2021), results were validated with a broader prior. Results with the broader prior can be found on DaRUS.

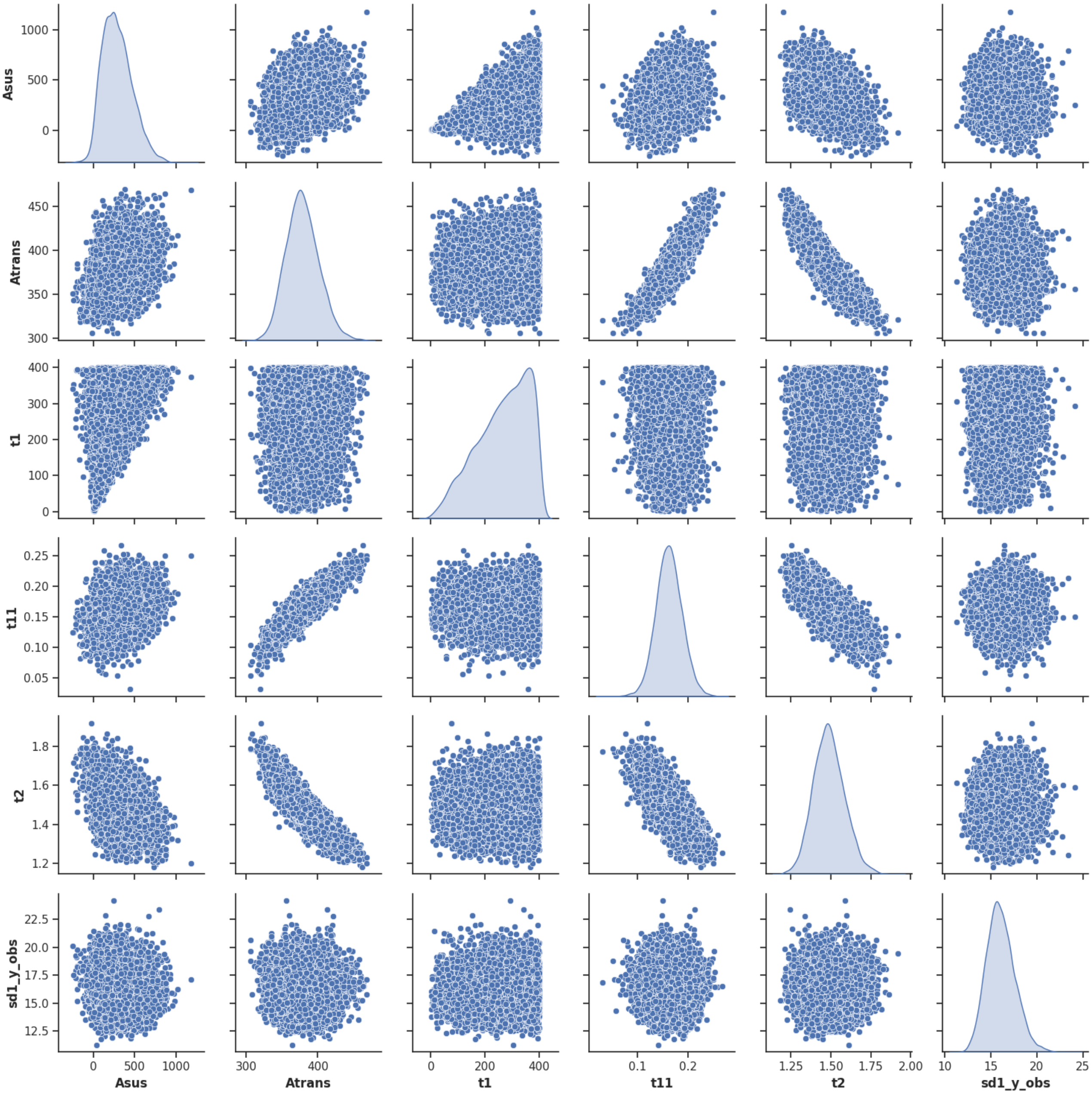

Fig. S 19. Posterior scatter plots of RTF model parameters for the codeine 4 weeks group in application example 3. 1D marginals and 2D parameter scatter plots from MCMC sampling of the posterior distribution for the codeine 4 weeks group. An immediate response ($T_{shift} = -2$) was assumed, and the initial drug concentration before injection was set to zero ($p_0 = 0$). T_{range} was set to the range of the experimental data ($T_{range} = 6$). The Effective Sample Size (ESS) was 33245.2 samples. According to the Bayesian Analysis Reporting Guidelines (Kruschke, 2021), results were validated with a broader prior. Results with the broader prior can be found on DaRUS.
